# Versatile MXene Gels Assisted by Brief and Low-Strength Centrifugation

**DOI:** 10.1007/s40820-023-01302-3

**Published:** 2024-01-22

**Authors:** Weiyan Yu, Yi Yang, Yunjing Wang, Lulin Hu, Jingcheng Hao, Lu Xu, Weimin Liu

**Affiliations:** 1 State Key Laboratory of Solid Lubrication, Lanzhou Institute of Chemical Physics, Chinese Academy of Sciences, Lanzhou, 730000 People’s Republic of China; 2 Shandong Laboratory of Advanced Materials and Green Manufacturing at Yantai, Yantai, 264006 People’s Republic of China; 3 Key Laboratory of Colloid and Interface Chemistry and Key Laboratory of Special Aggregated Materials, Shandong University, Jinan, 250100 People’s Republic of China

**Keywords:** MXene, Centrifugation-assisted rapid gelation, Lubrication, Supercapacitor, Anti-counterfeiting applications

## Abstract

Due to the mutual repulsion between their hydrophilic surface terminations and the high surface energy facilitating their random restacking, 2D MXene nanosheets usually cannot self-assemble into 3D macroscopic gels with various applications in the absence of proper linking agents. In this work, a rapid spontaneous gelation of Ti_3_C_2_T_*x*_ MXene with a very low dispersion concentration of 0.5 mg mL^−1^ into multifunctional architectures under moderate centrifugation is illustrated. The as-prepared MXene gels exhibit reconfigurable internal structures and tunable rheological, tribological, electrochemical, infrared-emissive and photothermal-conversion properties based on the pH-induced changes in the surface chemistry of Ti_3_C_2_T_*x*_ nanosheets. By adopting a gel with optimized pH value, high lubrication, exceptional specific capacitances (~ 635 and ~ 408 F g^−1^ at 5 and 100 mV s^−1^, respectively), long-term capacitance retention (~ 96.7% after 10,000 cycles) and high-precision screen- or extrusion-printing into different high-resolution anticounterfeiting patterns can be achieved, thus displaying extensive potential applications in the fields of semi-solid lubrication, controllable devices, supercapacitors, information encryption and infrared camouflaging.

**Supplementary Information:**

The online version contains supplementary material available at 10.1007/s40820-023-01302-3.

## Highlights

- A low-strength-centrifugation-assisted approach for rapid gelation of Ti_3_C_2_T_x_ MXene with a very low dispersion concentration into multifunctional 3D architectures is developed.
- On the basis of pH-induced surface termination changes, the MXene gels exhibit reconfigurable internal structures and tunable rheological, tribological, electrochemical, infrared-emissive and photothermal-conversion properties.
- By adopting a gel with optimized pH value, high lubrication, remarkable capacitance, long-term capacitance retention and high-precision screen- or extrusion-printing into high-resolution anticounterfeiting patterns can be achieved.

**Supplementary Information:**

The online version contains supplementary material available at 10.1007/s40820-023-01302-3.

## Graphical abstract

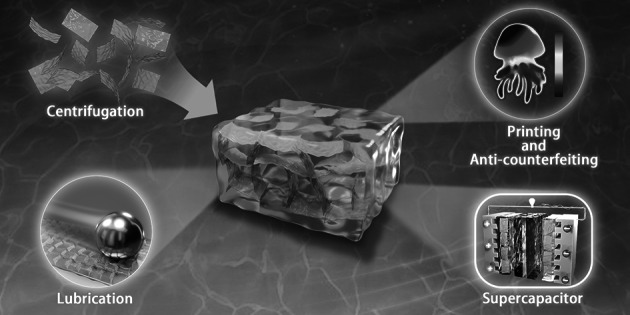

## Introduction

As an emerging family of 2D metal carbides or nitrides generally formulated with M_*n*+1_X_*n*_T_*x*_, where M, X and T represent transition metal, carbon/nitrogen and surface terminations of –F, –O and –OH, respectively, MXene has found a diverse range of potential applications in energy storage[1, 2], catalysis [3, 4], sensing [5, 6], lubrication [7, 8] and eco-friendly inks for printing and anti-counterfeiting techniques [9–11] in the past decade, thanks to its remarkable electrical conductivity, high surface hydrophilicity, tunable electronic properties, excellent tribological performance, low toxicity and good dispersibility in aqueous solutions. The major bottleneck problem that may negatively influence the performance of MXene-based materials is the random, irreversible restacking or aggregation of MXene nanosheets, which usually results in a profound attenuation in their accessible surface areas or ion diffusion capacities [4, 12, 13]. To address this issue, numerous strategies intending to assemble the 2D MXene nanosheets into a variety of 3D macroscopic architectures (including gels) have been developed [14–16].

Previous studies have shown that 2D inorganic nanomaterials can form gels when they self-assemble into interconnected 3D networks capable of holding a large quantity of water via electrostatic interaction, van der Waals association and hydrogen bonding [17–21]. Nonetheless, the presence of mutually repulsive –O and –OH terminal groups usually makes the adjacent MXene nanosheets hard to crosslink with each other. Hence, different kinds of binders such as divalent metal ions, graphene oxide (GO) and cationic surfactants or polymers are usually required to effectively overcome the electrostatic repulsive forces and link the nanosheets together [4, 12, 14, 22–25]. However, the introduction of additional organic or inorganic linkers suffers the risk of adversely altering the inherent properties such as electrical conductivity and lubricity of MXene nanosheets [4]. And the approaches relying on GO and organic molecules all involve a relatively complicated process where MXenes are inclined to be oxidized because of their high chemical activity [12]. Although demonstrated to be a fast and convenient strategy, the metal ions-assisted gelation is only accessible to an MXene dispersion with concentration above 5 mg mL^−1^ [12]. In other words, developing novel approaches for creating functional MXene gels at very low particle concentrations and mild conditions remains a great challenge [23, 26]. Moreover, MXene gels linked by different materials have found potentials applications in the fields of energy storage, catalysis, ink printing and thermal camouflage depending on their structures and properties [4, 12, 14, 22–25], but a facilely prepared and low-cost MXene gel with functional versatility that can be employed for multiple purposes has still seldomly been reported.

Here we illustrate a spontaneous gelation of Ti_3_C_2_T_*x*_ MXene into a water-rich (> 96 wt%) gel with functional versatility after only 30 s, 400 × g centrifugation with a dispersion concentration as low as 0.5 mg mL^−1^. On the basis of pH-induced variations in the surface chemistry of the nanosheet gelator and subsequent topological reconfigurations in its internal structure, the resultant gel exhibits excellent and controllable rheological, tribological, electrochemical, near-infrared (NIR)-emissive and photothermal-conversion properties that are comparable or even superior to the previously reported linkers-containing MXene gels. With optimized surface terminations and internal microstructures, the gel is testified to be an ideal candidate for high-performance semi-solid lubricants, supercapacitor electrodes and inks for printing and anti-counterfeiting applications, by virtue of its excellent friction and wear resistance, attractive specific capacitances, favorable capacitance retention, good viscoelasticity and thixotropy, large yield stress and tunable NIR emissivity and light-to-heat transition efficiency. Our study may therefore provide a simple and low-cost paradigm for creating novel MXene-based materials applicable to a variety of systems in materials science, colloidal chemistry, mechanical engineering, nanotechnology and tribology.

## Experimental Section

### Materials

Ti_3_AlC_2_ MAX powder was purchased from Jilin 11 Technology Co. Ltd, China. Lithium fluoride (LiF), ferrous chloride (FeCl_2_), zinc chloride (ZnCl_2_) and polydimethyldiallyammonium chloride (PDDA) aqueous solution (20 wt%) were all purchased from Shanghai Aladdin Biochemical Technology Co. Ltd, China. Hydrochloric acid (HCl, 36–38%) and sodium hydroxide (NaOH) were received from Sinopharm Chemical Reagent Co. Ltd, China. All the chemicals have a purity > 98% and were used as received. Ultrapure water (*ρ* = 18.25 mΩ cm) was used to prepare all the MXene dispersions and gels.

### Preparation of MXene Dispersions

In a typical procedure, 0.9 g Ti_3_AlC_2_ powder was mixed with 0.9 g LiF and 20 mL, 9 mol L^−1^ HCl under magnetic stirring at 35 °C for 24 h, followed by washing with iced deionized water under 10 min, 1200 × g centrifugation for several times until the pH of the supernatant reached ~ 6. Subsequently, the products were re-dispersed in 40 mL deionized water under alternate hand-shaking and sonication for 1 h and 5 min, respectively. The mixture was then centrifuged at 1500 × g for 1 h to obtain the MXene dispersion (10 mg mL^−1^).

The PDDA-modified MXene dispersion was prepared as follows [27]: First, aqueous solution of PDDA (10 mL, 1 wt%) was added dropwise into a 100 mL, 0.1 mg mL^−1^ MXene dispersion under magnetically stirring for 24 h; the mixture was then centrifuged at 1200 × g for 1 h and the resultant sediment was washed with deionized water under 1 h, 1200 × g centrifugation for three times; at last, the products were re-dispersed in deionized water and sonicated for 5 min to obtain a homogenous dispersion of PDDA-modified Ti_3_C_2_T_*x*_ nanosheets.

### Preparation of MXene Gels

The centrifugation-assisted MXene gels were prepared by centrifuging aqueous dispersions of Ti_3_C_2_T_*x*_ MXene (pH 10, ≥ 0.5 mg mL^−1^) at velocity above 400 × g for 30 s at ambient temperature. This process segregated the pristine uniform MXene dispersion into a MXene-rich sediment that was collected as the gel and a nanoparticle-free supernatant that was removed by pipetting. The MXene-PDDA composite gel was prepared using PDDA-modified Ti_3_C_2_T_*x*_ dispersions instead. The metal ions-assisted MXene gel was prepared by adding 200 μL, 1 mol L^−1^ FeCl_2_ or ZnCl_2_ solution into a 5-mL MXene dispersion with concentration above 5 mg mL^−1^ to a mass ratio of 3: 8 (metal chloride-to-MXene) in the absence of external centrifugal forces. Aqueous solutions (0.1 mol L^−1^) of NaOH and HCl were used to adjust the internal pH value of MXene gels.

### Characterization

A JEM-1011 transmission electron microscopy (TEM, JEOL, Japan) and a Multimode8 atomic force microscopy (AFM, Bruker, Germany) in tapping mode were used to characterize the microstructure of exfoliated Ti_3_C_2_T_*x*_ nanosheets. A JSM-7610F field-emission SEM (Zeiss, Germany) and a HITACHI SU3050 cryogenic SEM (cryo-SEM) were utilized to examine the internal microstructure of MXene gels. Samples for cryo-SEM observations were prepared by “sandwiching” a small amount of MXene gel between two gold planchettes. The “sandwich” was then quickly plunged into liquid ethane and transferred under vacuum into a nitrogen-cooled BAF060 freeze-fracture system (BalTec AG, Liechtenstein). At last, the sample was transferred to the precooled stage of SEM for observations using a BalTec VCT100 shuttle precooled with liquid nitrogen.

A ZetaNano ZS90 potential analyzer (Malvern, UK) was employed to measure the zeta potential of MXene dispersions at different pH values. A D8 Advance diffractometer (Bruker, Germany, Cu K*α*, *λ* = 0.154 nm) and a SAXSess MC2 high-flux SAXS instrument (Anton Paar, Austria, Cu K*α*, *λ* = 0.154 nm) were used to conduct XRD and SAXS analyses on lyophilized gel samples. A Escalab 250 Xi XPS spectrometer (Thermo Fisher, USA, Al Kα) was employed for XPS analyses with the C 1*s* peak at 284.6 eV used for calibrating the binding energy. A DXR2 Raman spectrometer (Thermo Fisher, USA) using a 633-nm laser as excitation source was utilized to obtain the Raman spectra of Ti_3_C_2_T_*x*_ gels at different pH values. A VERTEX-70/70v spectrometer (Bruker Optics, Germany) was used to carry out FTIR measurements in transmission mode. The lyophilized gel samples were stored in a desiccator to avoid moisture adsorption before the measurements. Spectra over 4000–400 cm^−1^ were obtained by taking 64 scans with a final resolution of 1 cm^−1^. An MCR-302 rheometer (Anton Paar, Austria) with a plate-plate system and a sample gap of 1.0 mm was employed to study the viscoelasticity, thixotropy and yield stress of the MXene gels. An ASAP 2460 instrument (Micromeritics, USA) was utilized to determine the N_2_ adsorption–desorption isotherms of the MXene gels at pH 4 and 10. The specific surface area was calculated according to the Brunauer–Emmett–Teller (BET) equation. A four-probe instrument (TH26011CS, China) was utilized to determine the electrical conductivity of a lyophilized aerogel monolith by repeating the measurement in at least ten different positions.

### Tribological Tests

An UMT-TRIBOLAB friction and wear tester (Bruker, Germany) with a reciprocating ball-on-disc configuration using the ZrO_2_ ball (*d* = 10 mm) and steel substrate (*d* = 24 mm) as counterparts was employed to measure the CoF of MXene gels at applied normal loads of 10, 30, 50 and 70 N and sliding velocity between 0.2 and 60 mm s^−1^. Both the ball and substrate were cleaned ultrasonically in petroleum ether and methanol before the measurements. A MicroXAM-800 3D noncontact surface profilometer (KLA-Tencor, USA) was used to detect the wear volume and 3D surface profile of a steel substrate after lubricated with different gels. All the tribological measurements were repeated for at least three times to ensure good reproducibility.

### Electrochemical Measurements

A CHI660E electrochemical workstation (Shanghai Chenhua, China) with a conventional three-electrode configuration was used to test the electrochemical performance of MXene gels that were adhered onto a glassy carbon current collector as the electrode directly. The measuring potential range was between − 1.1 and − 0.15 V for both the CV and CGD curves. Active carbon rod and Hg/Hg_2_SO_4_ were selected as the counter and reference electrodes, respectively, and 3 mol L^−1^ H_2_SO_4_ solution was used as the electrolyte. The EIS data were obtained in a range from 10 mHz to 100 kHz with a potential amplitude of 10 mV. The mass of adhered free-standing MXene gels was determined from the weight difference between an electrode before and after loading the gels. The amount of real active materials in an electrode was then calculated according to the water content of a MXene gel pre-determined from the weight difference of a gel before and after complete lyophilization. All the mass values were obtained from multiple repeated measurements. The mass loading of active materials in free-standing pH 4 and 10 MXene gel electrodes was measured to be 0.42 ± 0.07 and 0.38 ± 0.05 mg, respectively.

The specific capacitance based on the CV curves was calculated according to Eq. 1:

1 $$C = \frac{\smallint IdV}{{sVm}}$$

where *C* is the gravimetric capacitance (F g^−1^), *I* is the current (A) determined during the CV tests, *V* is the potential window (V), *s* is the scanning rate (V s^−1^), and *m* is the weight of active materials in an electrode (g).

The capacitance from the GCD curves was calculated by Eq. 2:

2 $$C = \frac{I\Delta t}{{m\Delta V}}$$

where *C* is the gravimetric capacitance (F g^−1^), *I* is the discharge current (A), Δ*t* is the discharge duration (s), *m* is the weight of active materials in an electrode (g), and Δ*V* is voltage change during the discharge process.

### Ink Printing and Anti-counterfeiting Tests

A LAMBDA 1050 + UV–VIS–NIR spectrometer (PerkinElmer, USA) equipped with an integrating sphere was used to measure the NIR emissivity of MXene gel inks at pH 4 and 10. In a typical screen-printing process, a certain amount of gel inks was spread onto a PET substrate that have been coated, cured and washed previously. Afterward, the inks were rapidly scraped across the patterns by a scraper. Alternatively, the inks can be directly extrusion-printed into different passwords or patterns on the substrate via a syringe. The thickness of the gel coatings can be regulated by multiple times printing. After natural drying, the anticounterfeiting performance was analyzed either photoemissively by heating the gel films with a HP-E1515 hot plate (Hanbang electronics, China) at 50 °C or photothermally by illuminating the ink coatings with an 808-nm FC-11 NIR laser (Changchun New Industries Optoelectronics Technology, China) at 3 W cm^−2^. A UTi120S thermal imager (Yuride, China) was used to detect the surface temperature and IR images of the screen- or extrusion-printed patterns.

## Results and Discussion

### Preparation and Characterization of the MXene Gel

**Fig. 1:**
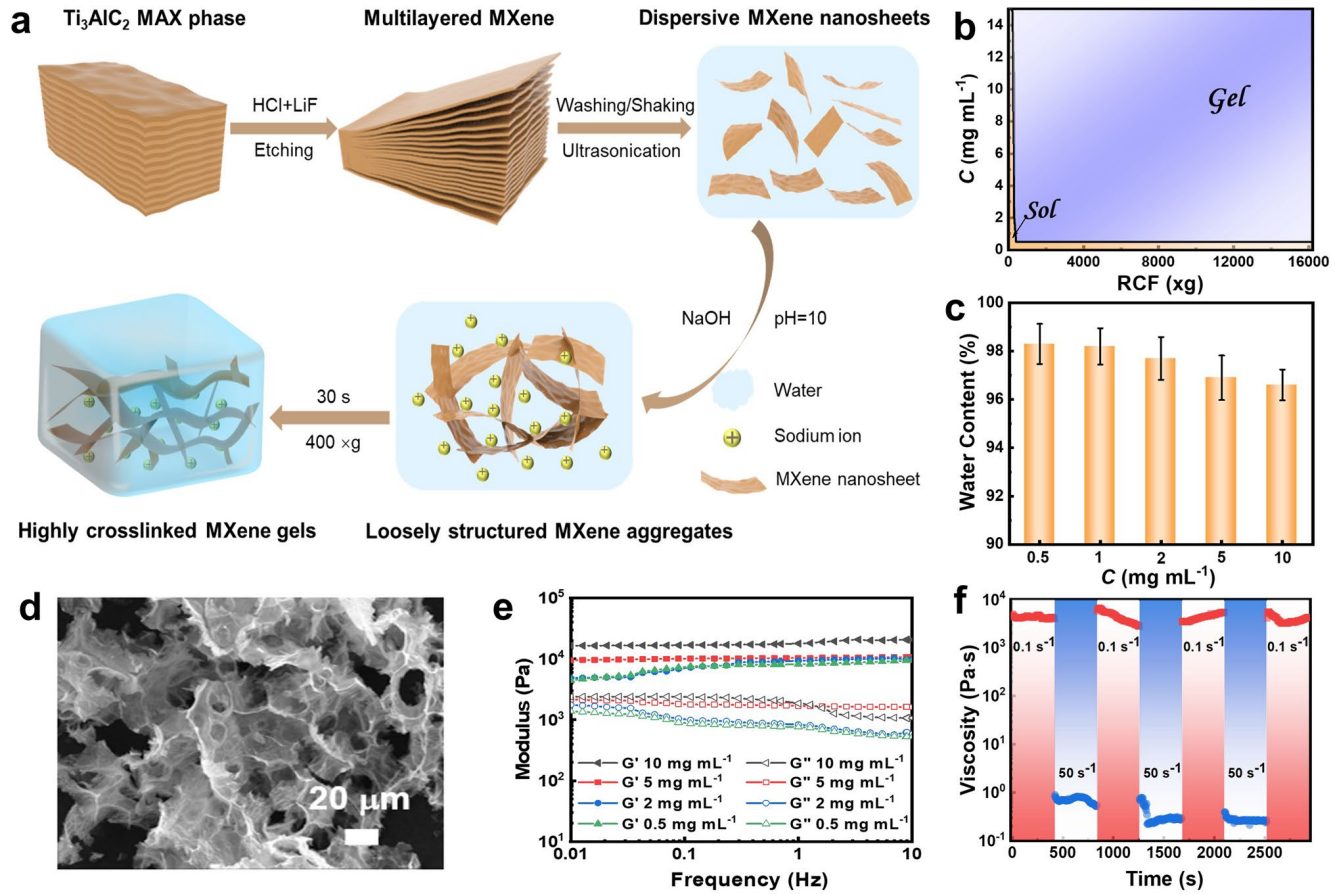
Preparation and characterization of the MXene gel. **a** Schematic illustration of producing Ti_3_C_2_T_*x*_ MXene gelators and centrifugation-assisted MXene gels. **b** Phase diagram of different-concentration pH 10 Ti_3_C_2_T_*x*_ dispersions after exposure to different relative centrifugal forces (RCFs). The blue-colored region refers to a particle concentration and RCF required for obtaining a gel. **c** Water content of MXene gels prepared with Ti_3_C_2_T_*x*_ dispersions in different concentrations. **d** Scanning electron microscopy (SEM) observation of a MXene gel formed with a dispersion concentration of 10 mg mL^−1^. **e** Rheological property of the gels prepared with different initial particle concentrations. **f** Thixotropic property of the gel formed with a dispersion concentration of 10 mg mL^−1^. *T* = 25 °C. (Color figure online)

Ultrathin MXene nanosheets (~ 1.5 nm in thickness and 1.2 ± 0.3 μm in lateral size) terminated with –F, –O and –OH groups were prepared by LiF/HCl etching of bulk layer-structured Ti_3_AlC_2_ MAX powders followed by proper washing, shaking and ultrasonication as schematically illustrated in Fig. 1a [5, 12, 14]. Their microstructures and morphology were characterized by the transmission electron microscopy (TEM) and atomic force microscopy (AFM) observations (Fig. S1a–d) and were found to be prominently different from the pristine MAX phase and the multilayered MXene obtained after etching (Fig. S1e, f). They all had a smooth surface free of any oxide particles, and their thickness was consistent with previous reports [28–30]. The results thus indicate a successful production of high-quality monolayered Ti_3_C_2_T_x_ MXene nanosheets that were able to act as effective building blocks for various 3D MXene architectures [28–30]. Zeta potential measurements in Fig. S2 reveal that the colloidal stability and interparticle electrostatic repulsion of Ti_3_C_2_T_*x*_ nanosheets both attained highest at pH 10 with a value of − 39.5 ± 1.59 mV [31, 32].

A few previous works including our own have certified that an aqueous dispersion of charged inorganic nanosheets sometimes exhibits gel-like behavior once the nanosheets are constrained close to each other by external centrifugal forces so that their mutual electrostatic repulsion can be sufficiently strong to confine their mobility in the system [17, 20, 21, 33, 34]. To examine whether this approach is also applicable for overcoming the repulsive forces between MXene nanosheets and inducing their gelation, pH 10 Ti_3_C_2_T_*x*_ particles with a highest electronegativity were used as a representative MXene gelator in this study. In a typical procedure (see details in Experimental Section), centrifugation at an optimal external force and nanoparticle concentration could rapidly (within 30 s) segregate a pristine, homogenous MXene dispersion into a gel-like sediment and a Ti_3_C_2_T_*x*_-free supernatant that can be easily removed via pipetting. Phase diagram in Fig. 1b and photographs in Figs. S3 and S4 demonstrate that the centrifugation-induced MXene gels can be acquired with a very low initial particle concentration of 0.5 mg mL^−1^ when the centrifugal force reached 400 × g. By contrast, as suggested in Figs. S5a, b, metal ions such as Fe^2+^ and Zn^2+^ cannot induce gelation of pH 10 Ti_3_C_2_T_*x*_ MXene at a particle concentration lower than 5 mg mL^−1^ in absence of external centrifugal forces. And an aqueous dispersion containing 2 mg mL^−1^ Ti_3_C_2_T_*x*_ MXene and 5 mmol L^−1^ Fe^2+^ can be transformed to gels upon exposure to 400 × g centrifugation for 30 s (Fig. S5c). The results thus verify the effectiveness and dominant role of external low-strength centrifugal forces in triggering rapid gelation of Ti_3_C_2_T_*x*_ MXene at low particle concentrations.

Figure 1c demonstrates that the as-prepared MXene gels using different initial Ti_3_C_2_T_x_ concentrations all had a high water content between 96.5 and 98.2 wt%. And changing the external centrifugal force in a broad range between 400 and 16,000 × g only caused the water content to vary within 1 wt%. A large area of highly crosslinked 3D networks can be discovered in a freeze-dried gel as displayed in the SEM imaging in Figs. 1d and S6a. On the contrary, direct lyophilization of the pristine Ti_3_C_2_T_*x*_ dispersion only resulted in relatively loosely structured aggregates (Fig. S6b). The results therefore suggest that the spontaneous interconnection between Ti_3_C_2_T_*x*_ nanosheets into a swelling gel network was essentially triggered by the applied centrifugal forces as schematically illustrated in Fig. 1a. Figure S7 further demonstrates that the lyophilization essentially transformed a MXene gel into an aerogel monolith as no prominent collapses or fractures in the 3D macrostructures could be detected after freeze-drying. Cryogenic SEM observations (Fig. S8) also show a formation of highly crosslinked networks with developed numerous pore structures inner a pristine MXene gel. Rheological measurements in Fig. 1e show that the MXene-rich sediments could maintain a storage modulus (*G*′) > loss modulus (*G*′′) behavior over a broad sweeping frequency range from 0.01 to 10 Hz, which was indicative of their gel-like natures. And the gels prepared with different dispersion concentrations all yielded *G*′ and *G*′′ values at around 10^4^ and 10^3^ Pa, respectively, due to their very close water contents. An excellent thixotropic performance of the centrifugation-assisted MXene gel was revealed in Fig. 1f, in which its viscosity could exhibit fast response and recovery upon alternately changing external shear rates. It should be noted that although possessing an equal water content of ~ 97 wt%, the Fe^2+^- and Zn^2+^-assisted MXene gels displayed shear moduli that were more than one order of magnitude lower than those of a centrifugation-induced gel (Fig. S9). The prominently improved viscoelasticity and splendid thixotropic property could not only enable the MXene gel to serve as inks for high-precision screen- or extrusion-printing [35, 36], but also as semi-solid lubricants that usually require potent deformation- and creep-recoiling capacities [37, 38].

The formation of loosely structured MXene aggregates in Fig. S6b suggests that the presence of Na^+^ cations may screen the electrostatic repulsion between adjacent MXene nanosheets and consequently lead to weak association or crosslinking between them. This can be confirmed by Fig. S10a, in which pH 10 MXene dispersions prepared with other alkalis, i.e., KOH, NH_4_OH and (C_4_H_9_)_4_NOH can all afford gels after an identical short and low-strength centrifugation, and the addition of (C_4_H_9_)_4_NOH can even cause coagulation of Ti_3_C_2_T_*x*_ nanosheets before centrifugation. It demonstrates that univalent cations including Na^+^, K^+^, NH_4_^+^ and (C_4_H_9_)_4_N^+^ were able to attenuate the inter-particle repulsion and the (C_4_H_9_)_4_N^+^ was more effective in neutralizing negative charges according to the Hofmeister series. SEM observations in Fig. S10b further show that the resultant MXene gels all had similar highly crosslinked internal network structures. Previous study by Yang et al. has demonstrated that divalent or trivalent metal ions such as Fe^2+^ and Al^3+^ can effectively trigger fast gelation of Ti_3_C_2_T_*x*_ MXene in the absence of external forces at a particle concentration above 5 mg mL^−1^, while univalent cations (e.g., K^+^) cannot due to its relatively low hydration energy [12]. Figure S5c shows that divalent Fe^2+^ at a concentration equal to the univalent Na^+^ (~ 5 mmol L^−1^) also cannot induce gelation of pH 10, 2 mg mL^−1^ Ti_3_C_2_T_*x*_ MXene unless the mixture was exposure to external 400 × g centrifugal forces. In addition, Zhang et al. and Fan et al. have shown that a MXene dispersion without pH control and addition of alkaline can be transformed into highly concentrated inks after centrifugation at a sufficiently high speed and long period [39–41]. Hence, the applied external centrifugal forces should play a more dominant role than the cations in triggering rapid gelation of Ti_3_C_2_T_*x*_ MXene at very low particle concentrations.

Building on our findings in Figs. 1 and S1–S10, the mechanism for low-strength centrifugation-assisted fast gelation of Ti_3_C_2_T_*x*_ MXene can be summarized as follows: First, the Na^+^ ions in the pristine aqueous dispersion screened the electrostatic repulsion between MXene nanosheets at relatively high pH values and induced formation of loosely structured aggregates. Such metastable weakly associated or crosslinked MXene precursors were then kinetically entrapped into a gel-like sediment with the mobility of MXene nanosheets strongly restricted and eventually grew into highly crosslinked network structures after exposure to external centrifugal forces. Corresponding schematic illustration is displayed in Fig. 1a.

**Fig. 2:**
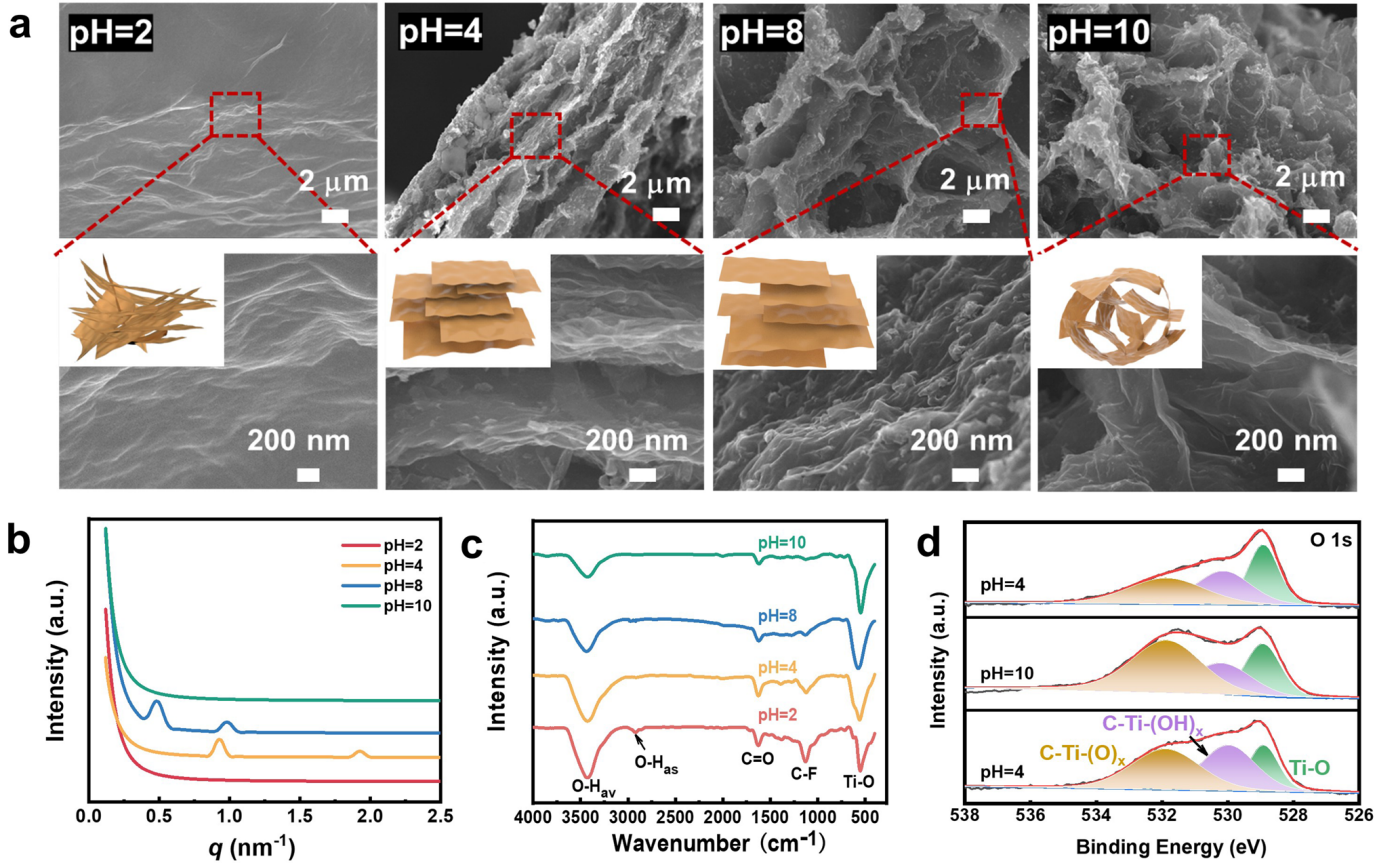
pH-dependent internal structures of the MXene gel. **a** SEM images, **b** SAXS profiles, **c** FTIR spectra and **d** XPS O 1*s* patterns of freeze-dried Ti_3_C_2_T_*x*_ gels with different internal pH values. Insets in **a** are schematic illustrations of the aggregation behavior of MXene nanosheets inside a gel. *T* = 25 °C

SEM observations in Fig. 2a depict a pH-induced internal nanosheets rearrangement of the MXene gel, although such microstructural changes would not cause significant variations in its mechanical elasticity (Fig. S11). This microstructure-independent elasticity was similar to the previously reported aqueous graphene oxide gels [42]. It should be noted that all the rheological measurements in this work were commenced straight after loading the sample and the viscoelasticity of MXene gels did not display significant variations over time as illustrated in Fig. S12. Small-angle X-ray scattering (SAXS) data in Fig. 2b show that the Ti_3_C_2_T_*x*_ particles reorganized into layer-by-layer configurations with two Bragg diffraction peaks detected at *q*_1_ =  ~ 0.49 and *q*_2_ =  ~ 0.98 nm^−1^, respectively, as the pH of the gel lowered from 10 to 8, corresponding to the formation of long-range ordered lamellar architectures with a uniform periodicity of ~ 12.8 nm (*d* = 2*π*/*q*) [17, 20, 21]. This set of diffraction peaks exhibited a profound shift to large angles upon further reducing the pH to 4, owing to shrinkage of the interlamellar spacing to ~ 6.54 nm [21]. The nanosheets became closely and randomly arrayed with no measurable diffractions as the pH value reached as low as 2. Raman spectra in Fig. S13a show that all the gel samples exhibited two weak I and II bands related to Ti–C at around 400 and 600 cm^−1^ as well as D-(disordered) and G-(graphite) bands at ~ 1400 and ~ 1590 cm^−1^, respectively. The intensity ratio of the D and G bands attained as high as 0.87 and 0.94 at pH 8 and 4, indicative of a high degree of graphitization and orientational ordering of Ti_3_C_2_T_*x*_ MXenes as a consequence of formation of lamellar phases [43], whereas it reached a minimum of 0.68 at pH 10 due to the presence of amorphous interconnected network structures [43]. The signature (002) peak of MXene can be traced in the X-ray diffraction (XRD) patterns for all the gel samples (Fig. S13b), which suggests that the pH variations did not disrupt the inherent crystalline structure of Ti_3_C_2_T_*x*_ nanosheets [23]. A shift in the peak position to large angles of the pH 4 gel compared to that of the pH 8 one also demonstrates a shortening of interlamellar distance among the MXene layers [23]. Figures S10 and S14 show that such internal structural changes can be achieved using other alkalis and acids including KOH, NH_4_OH, (C_4_H_9_)_4_NOH, H_2_SO_4_ and HNO_3_ for regulating the internal pH value of a MXene gel other than NaOH and HCl, resulting in nearly identical microstructures at both relatively high and low pH values.

Figure 2c illustrates that a lyophilized MXene gel displayed major vibration bands ascribed to O–H, C=O, C–F and Ti–O at around 3425, 2940, 1640, 1130 and 560 cm^−1^, respectively. Both the intensity ratio of *v*_O–H_ to *v*_Ti–O_ and *v*_C–F_ to *v*_C=O_ prominently attenuated as a function of pH, possibly due to an alkali-induced deprotonation of the –OH groups and conversion of fluorines into oxygens at the surface terminations [23, 43, 44]. Such findings can be confirmed by the X-ray photoelectron spectroscopy (XPS) profiles in Figs. 2d and S15, where the intensity of the absorption bands correlated to –O terminal groups at around 532 eV significantly enhanced while that of the bands from –OH or –F terminations at around 530 and 685 eV, respectively, strongly reduced upon elevating pH from 4 to 10. Quantitative analysis in Table S1 further shows that the content of –O termination, determined by the deconvolution and integration of corresponding band areas in XPS spectra, improved from 23.9 to 48.0% with pH rising from 4 to 10. By contrast, the contents of –F and –OH terminal groups lowered from 50.4% and 25.7% to 30.3% and 21.7%, respectively. Zeta potential measurements in Fig. S2 indicate a highly strengthened electrostatic repulsion between Ti_3_C_2_T_*x*_ nanosheets under basic conditions, since their potential values nearly doubled with pH rising from 2 to 10 [31]. A hypochromatic shift by ~ 10 cm^−1^ in the band position of *v*_*av*O–H_, which corresponds to disruption of the hydrogen bonding between terminal hydroxyl groups [20, 45], can also be discovered in the Fourier transform infrared (FTIR) spectra in Fig. 2c as pH changed from 2 to 10. Figures 2d, S15, S16 and Table S1 further show that such transitions in surface chemistry of Ti_3_C_2_T_*x*_ MXenes were reversible, since both the FTIR and XPS absorptions of pristine pH 4 gel can be recovered after an alternate alkaline and acid treatments. Similar findings can be discovered in Hong et al.’s work [46], where the –F terminations of Ti_3_C_2_T_*x*_ MXene can gradually evolve to –OH and then to –O after an alkali-induced treatment, and the resultant Ti_3_C_2_(OH)_*x*_ and Ti_3_C_2_O_*x*_ powders regained a relatively high amount of –F groups after re-submerged in HF solutions. Hence, the pH-controlled internal structural alterations of MXene gel can be attributed to an interplay between the protonation/deprotonation of –OH groups and reversible conversion between the terminal –F and –O, which determined the strength between the competitive hydrogen bonding associations and mutual electrostatic repulsions among the nanosheets. A control experiment in Fig. S17 shows that addition of an equal amount of NaCl instead of NaOH to a pH 4 gel cannot reproduce the microstructure of a pH 10 gel, thus verifying that the internal topological reconfigurations were essentially caused by changes in pH rather than ionic strength. It is worthwhile to note that although all the gel samples used in this study were all prepared with a dispersion concentration of 10 mg mL^−1^, however, due to the addition of HCl/NaOH for adjusting pH and formation of internal microstructures with different packing densities, the final particle concentration in the gels with different pH values can be slightly different between 3 and 5 wt%; such minute variations in MXene concentration should not cause prominent alterations in the gel properties.

### Tribological Property of the MXene Gel

**Fig. 3:**
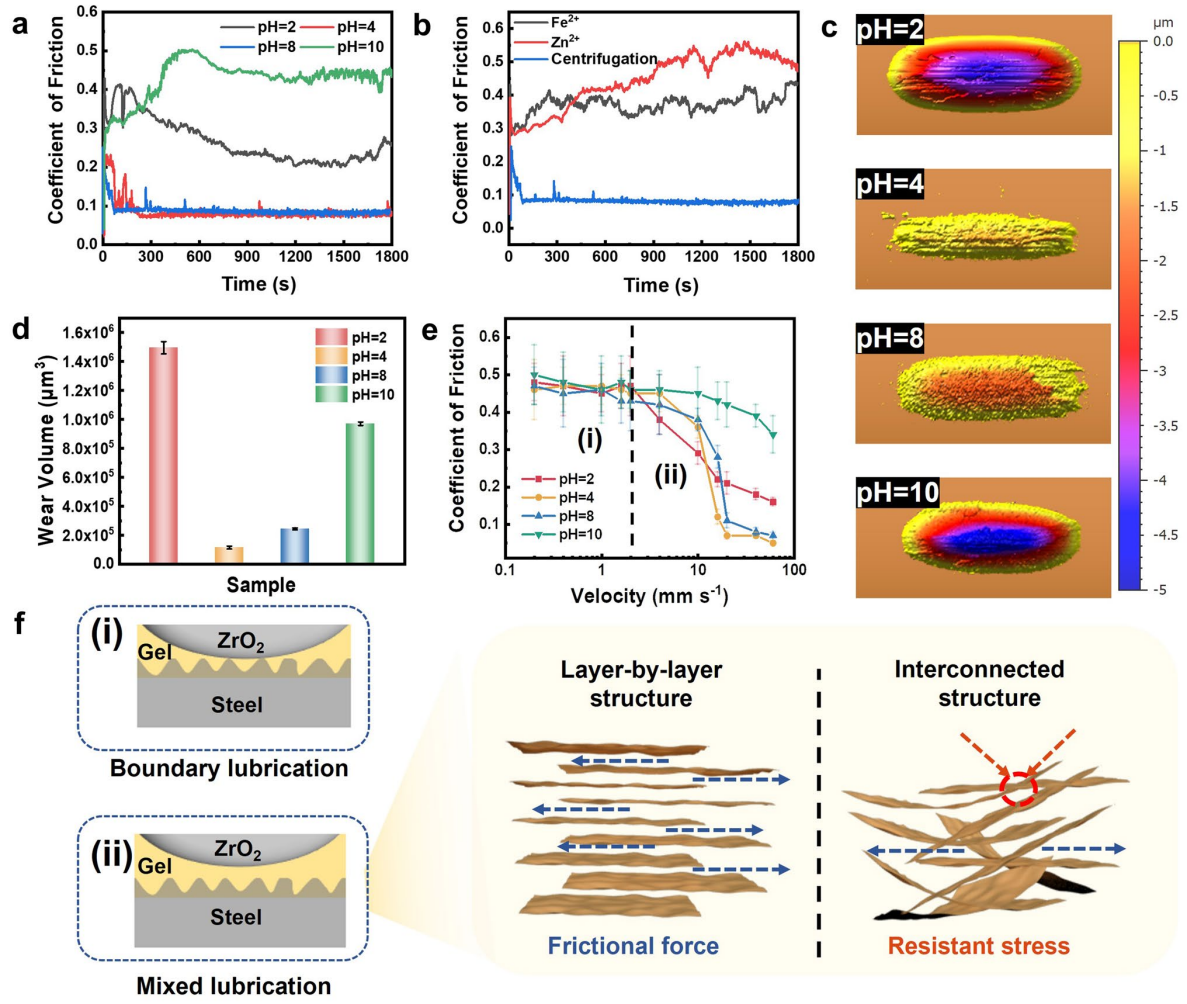
Tribological property of the MXene gel. **a** CoF of Ti_3_C_2_T_*x*_ gels with different pH values for the steel/ZrO_2_ tribopair at 10 N and 10 mm s^−1^. **b** Comparison between the CoF of centrifugation- and metal ions-induced MXene gels. **c** 3D surface topography and **d** abrasive volume of the steel substrate lubricated with Ti_3_C_2_T_*x*_ gels in different pH values. **e** Effect of sliding velocity on the CoF of MXene gels at a constant load of 10 N. **f** Schematic illustrations of the impact of sliding velocity and internal microstructure on the lubrication mechanism. *T* = 25 °C

Semi-solid lubricants (e.g., gels and greases) are advantageous to traditional liquid lubricants in their excellent mechanical strength, anti-creeping and creep-recoiling properties [47–49]. And the use of a purely inorganic gel lubricant can further reduce the environmental burdens and safety concerns caused by the presence of flammable and toxic organic base oils and additives such as mineral oils and thickeners [20, 21]. To probe whether the MXene gel can also function as an effective semi-solid lubricant, we tested its tribological behavior at different pH values for the steel/ZrO_2_ tribopair under 10-N applied normal load and 10 mm s^−1^ sliding velocity. Corresponding experimental results are displayed in Fig. 3a. It can be seen that the gel exhibited relatively high coefficients of friction (CoF) of ~ 0.25 and ~ 0.45, respectively, at the lowest and highest studying pH values, whereas it can provide CoF as low as ~ 0.08 that indicates a good lubricity at intermediate pH values (i.e., 4 and 8). Comparative measurements in Fig. 3b show that a pH 8 MXene gel initiated by Fe^2+^ or Zn^2+^ exhibited largely deteriorated lubrication with CoF reaching up to ~ 0.39 and ~ 0.51, respectively. Figure S18 demonstrates a potent load-carrying capacity of the Ti_3_C_2_T_*x*_ gel lubricant, given that its low CoF can be retained with external normal load attaining as high as 70 N. And the gels prepared with different dispersion concentrations all had an identical anti-friction capability. Although having negligible differences in the CoF, the pH 4 MXene gel was testified to possess a preferable abrasion resistance to the pH 8 one as it can provide much smaller abrasive scars and twofold lower wear volumes for a steel substrate as illustrated in Figs. 3c, d and S19.

Figure 3e depicts the relationship between the CoF of MXene gels and sliding velocity at a constant normal load of 10 N. As shown, at velocity below 2 mm s^–1^, the gels in different pH values all yielded a nearly invariable CoF at around 0.45, featuring a typical boundary lubrication where the gels preserved their nonflowable, solid-like behavior with no hydration layer generated at the metal/ceramic interface (Fig. 3f(i)). At velocity above 2 mm s^–1^, the CoF of gels declined versus sliding velocity, corresponding to transition of lubrication mechanism into mixed lubrication, in which the gel networks collapsed and resulted in a liquid hydration layer capable of providing relatively high lubricity in the contact area as schematically illustrated in Fig. 3f(ii). We did not employ a sliding velocity higher than 60 mm s^–1^ due to the upper measuring limit of the friction and wear tester used in this study. Figure S20 displays that the gel can reversibly convert its lubrication mechanism between boundary and mixed lubrications for several cycles without significant changes in its CoF, thus testifying its efficacy as an effective semi-solid lubricant that can act as a liquid lubricant to provide excellent friction and wear resistance for metallic materials upon exposure to strong external sliding forces. And it was able to restore its native solid-like behavior to furnish splendid anti-creeping property once the frictional forces were highly reduced or removed. A superior lubricity of gels with internal lamellar microstructures during mixed lubrication was likely because, on the one hand, the weak interlayer associations may facilitate fracture separations to take place at the face-to-face region that consequently makes the MXene nanosheets easy to shear [20, 50], and on the other hand, the formation of randomly arrayed, interconnected structures may introduce additional shear resistance at the stagger site [51]. Corresponding schematic diagram of such structural influence is displayed in Fig. 3f. Figure S21 shows that Ti_3_C_2_T_*x*_ MXene modified with a cationic ligand polydimethyldiallyammonium (PDDA) could produce gels with layer-by-layer and interconnected internal architectures at pH 2 and 8, respectively. And the PDDA-containing gel was found to provide much lower CoF and much smaller wear tracks at pH 2, thus verifying the profound impact of internal nanosheets configuration on the tribological performance of a MXene-based gel.

Although MXene has been employed as effective additives for improving the functionality of conventional organic-based semi-solid lubricants, to date, there have been no reports on gel lubricants entirely consisting of MXene nanosheets and water. Here we have achieved organic-free MXene gel lubricants with the aid of external centrifugal forces. The lubricity of resulting gels was also comparable to that of the gel lubricants from MXene-organic molecule hybrids, which had similar CoF at around 0.1 [52–54]. As a consequence, our work may provide new insights into developing new-generation eco-friendly semi-solid lubricants that are thoroughly free of any flammable and toxic organic base oils and additives. In addition, the pH-controlled lubrication may further enable the MXene gel to serve as a versatile lubricant allowing on-demand control over the tribological interaction between two contacting moving parts of a machine or device [55, 56]. For instance, it can effectively reduce the friction and abrasion of the moving parts of both a large equipment and small device that contributes to prolonging their service life at intermediate pH values. And on the contrary, it can provide relatively high friction to maintain the operations of a machine at desired accuracy, efficiency and reliability at both a low and high pH values. Such property is crucial for developing advanced technologies such as smart devices and surfaces, microfluidic technology, lab-on-chip systems and controllable transportation systems [20, 55–57].

### Electrochemical Performance of the MXene Gel

**Fig. 4:**
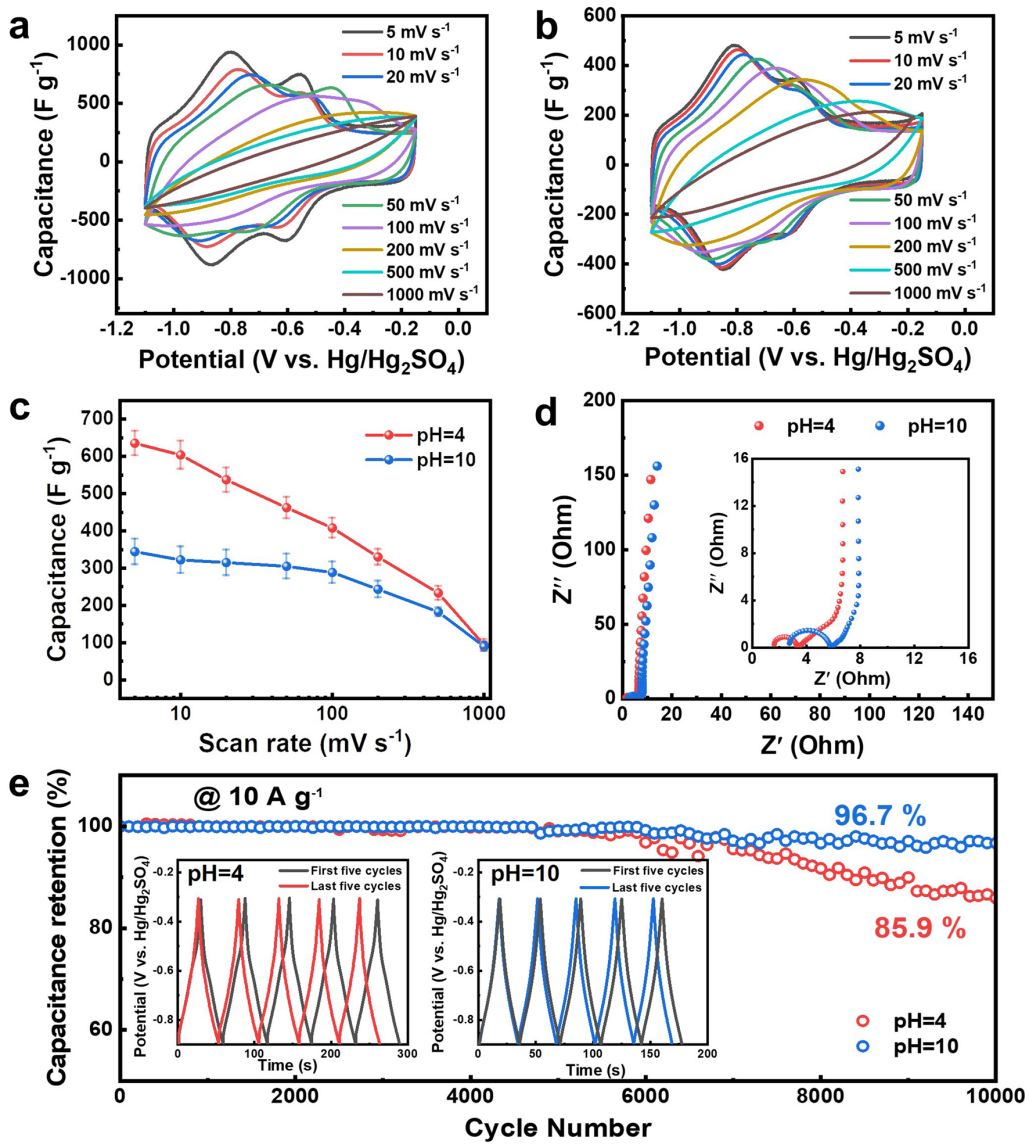
Electrochemical performance of the MXene gel electrode in 3 mol L^−1^ H_2_SO_4_ electrolyte. CV curves of **a** pH 4 and **b** 10 MXene gels at various scanning rates. **c** Specific capacitance of the pH 4 and 10 gels at different scanning speeds. **d** Electrochemical impedance spectroscopy (EIS) data and **e** capacitance retention tests of the gel electrodes at 10 A g^−1^. Insets in **d** and **e** are the Nyquist plots at high-frequency range and the GCD curves for the first and last five cycles, respectively. *T* = 25 °C

The electrochemical performance of the centrifugation-assisted MXene gel was evaluated to probe its potential applications as a freestanding electrode for supercapacitors. The advantages of using such freestanding gel electrodes include their facile fabrication and no use of inactive binders or conductive additives [12]. Representative photographs of an electrode produced from the centrifugation-assisted Ti_3_C_2_T_*x*_ gel are displayed in Fig. S22. Figure S23 evidences that the gel could maintain a long-term structural stability with no collapse or swelling occurring after immersed in a water reservoir for about one month, thus being suitable for directly used as a free-standing electrode without drying into an aerogel or printing into a pattern [58]. The water content before and after immersion was determined to be 96.4 ± 0.9 wt% and 96.9 ± 0.7 wt%, respectively. Figure 4a, b shows that the gel yielded cyclic voltammetry (CV) curves with two pairs of redox peaks detected at ~ –0.9 and –0.6 V (vs*.* Hg/Hg_2_SO_4_) in the potential range between –1.1 and –0.15 V at both pH 4 and 10, featuring a signature pseudo-capacitive behavior of Ti_3_C_2_T_*x*_ MXene [2]. The redox peaks can be reserved as the scanning rate attained as high as 200 mV s^–1^, indicative of a high surface utilization of Ti_3_C_2_T_x_ nanosheets and a good rate capability [12]. Figure S24 displays the galvanostatic charge–discharge (GCD) profiles of the Ti_3_C_2_T_*x*_ gel at current densities between 0.1 and 50 A g^–1^, where the charge/discharge curves for both pH 4 and 10 gels were nonlinear with no presence of apparent potential platforms, suggestive of a synergistic contribution of pseudo- and double-layer capacitances. The discharge time of a pH 4 gel was also much longer than that of a pH 10 gel at the same current density, corresponding to its much higher specific capacitance, which was consistent with the experimental results of the CV curves. Figure 4c reveals a remarkable specific capacitance at between 635.2 ± 34.3 and 407.9 ± 29.1 F g^–1^ for the pH 4 MXene gel in a scanning rate range from 5 to 100 mV, which consequently exhibits a massive potential for creating high-performance energy storage materials, as well as a satisfactory rate capability as it was able to produce a capacitance of 233.1 ± 11.7 F g^–1^ even when the scanning rate attains as high as 500 mV. While excellent capacitances such as 322.1 ± 37.6 F g^–1^ at 10 mV s^–1^ and 288.4 ± 26.9 F g^–1^ at 100 mV s^–1^ can also be obtained when using the pH 10 Ti_3_C_2_T_*x*_ gel with a strongly improved rate performance, given its capacitance did not fall significantly over a broad scanning speed range from 5 to 500 mV. It is worthwhile to note that the specific capacitance of pH 4 MXene gel (e.g., 635.2 ± 34.3 F g^–1^ at 5 mV s^–1^ and 603.7 ± 35.7 F g^–1^ at 10 mV s^–1^) was very likely to be the highest among the MXene gel electrodes using the same three-electrode cell configuration with the same Hg/Hg_2_SO_4_ reference electrode and H_2_SO_4_ electrolyte reported so far. It was also exceedingly comparable to a list of other previously reported MXene-gel-based electrodes with different reference electrodes and electrolytes as summarized in Table S2.

The Nyquist plot of pH 4 MXene gel shows a much smaller semicircle in the high-frequency region relative to that of the pH 10 gel in Fig. 4d, which represents a facile charge transport and smaller interfacial contact resistance positively contributing to a profoundly improved capacitance [59]. On the contrary, it also shows a relatively longer 45° line (i.e., the so-called Warburg type-line) corresponding to reduced reactive sites accessible in a brief period than that of the pH 10 gel, which may lead to deteriorated rate capability [59]. Four-probe measurements on lyophilized aerogel monoliths uncover a ultrahigh electrical conductivity at ~ 20,400 m S^–1^ of the pH 4 MXene architecture with relatively closely packed internal layer-by-layer structures, which is exceedingly comparable to and surpass a number of previously reported MXene-based aerogels (Table S3) detected using the same method, while a pH 10 Ti_3_C_2_T_*x*_ aerogel having relatively loosely structured internal porous networks yielded a significantly lower conductivity ~ 3,800 m S^–1^. Similar findings have been discovered in previously reported graphene-based capacitive electrodes, in which relatively densely packed nanosheet assemblies could lead to a relatively lower resistance and higher conductivity [58]. The N_2_ adsorption/desorption measurements in Fig. S25a show that both the pH 4 and 10 gels exhibited characteristic type-IV isotherms with prominent adsorption hysteresis loops between 0.5 and 1 and between 0.1 and 1 relative pressures, respectively, indicating the presence of numerous mesopores. The pore size was determined to be ~ 2 and ~ 3.9 nm for the gels at pH 4 and 10, respectively, as displayed in Fig. S25b. The specific surface area was measured to be ~ 10.2 and ~ 35.7 m^2^ g^–1^ for the pH 4 and 10 gels, respectively. The facile charge transport and ultrahigh conductivity may lead to the splendid specific capacitance of the pH 4 MXene gel, whereas the lower rate capability of a pH 4 MXene gel relative to that of a pH 10 gel may be due to, on the one hand, the threefold lower surface area reducing the surface utilization and available active sites of Ti_3_C_2_T_*x*_ MXene, on the other hand, a smaller interlayer spacing between MXene nanosheets (Figs. 2b and S13b) and a declined pore size and distribution restricting a fast ion diffusion [59, 60].

Figure 4e illustrates an excellent cyclic performance of the pH 10 MXene gel with a 96.7% capacitance retention after 10,000 cycles. The results are comparable to a list of previously reported MXene-based 3D macrostructures (Table S2). And the shape of the GCD profiles for the initial and last five cycles was nearly identical to each other. In comparison, although possessing a much higher capacitance, the cyclic stability of a pH 4 gel was good but relatively lower with an 85.9% retention in its capacitance over the same period. The GCD curves of the first and last five cycles also exhibited certain differences. Our results thus suggest a pH-controlled electrochemical performance of the linkers-free MXene gel, which can be utilized to fabricate advanced supercapacitors capable of providing either exceptional capacitances with satisfactory longevity or both splendid capacitances and cyclic stability depending on the concrete working conditions. The relatively lower cyclic stability of a pH 4 MXene gel in comparison with that of a pH 10 gel may be an interplay between the layer-by-layer arrangement of Ti_3_C_2_T_*x*_ nanosheets at pH 4 introducing volumetric deformation that can generate osmotic stress to crack, pulverize or detach active materials in an electrode during the long and repetitive charge–discharge cycles [61] and the formation of numerous porous structures at pH 10 providing sufficient free spaces that can buffer the volumetric variations of active materials during the cyclic measurements at a relatively large current density [62].

### Ink Printing and Anti-Counterfeiting Applications

**Fig. 5:**
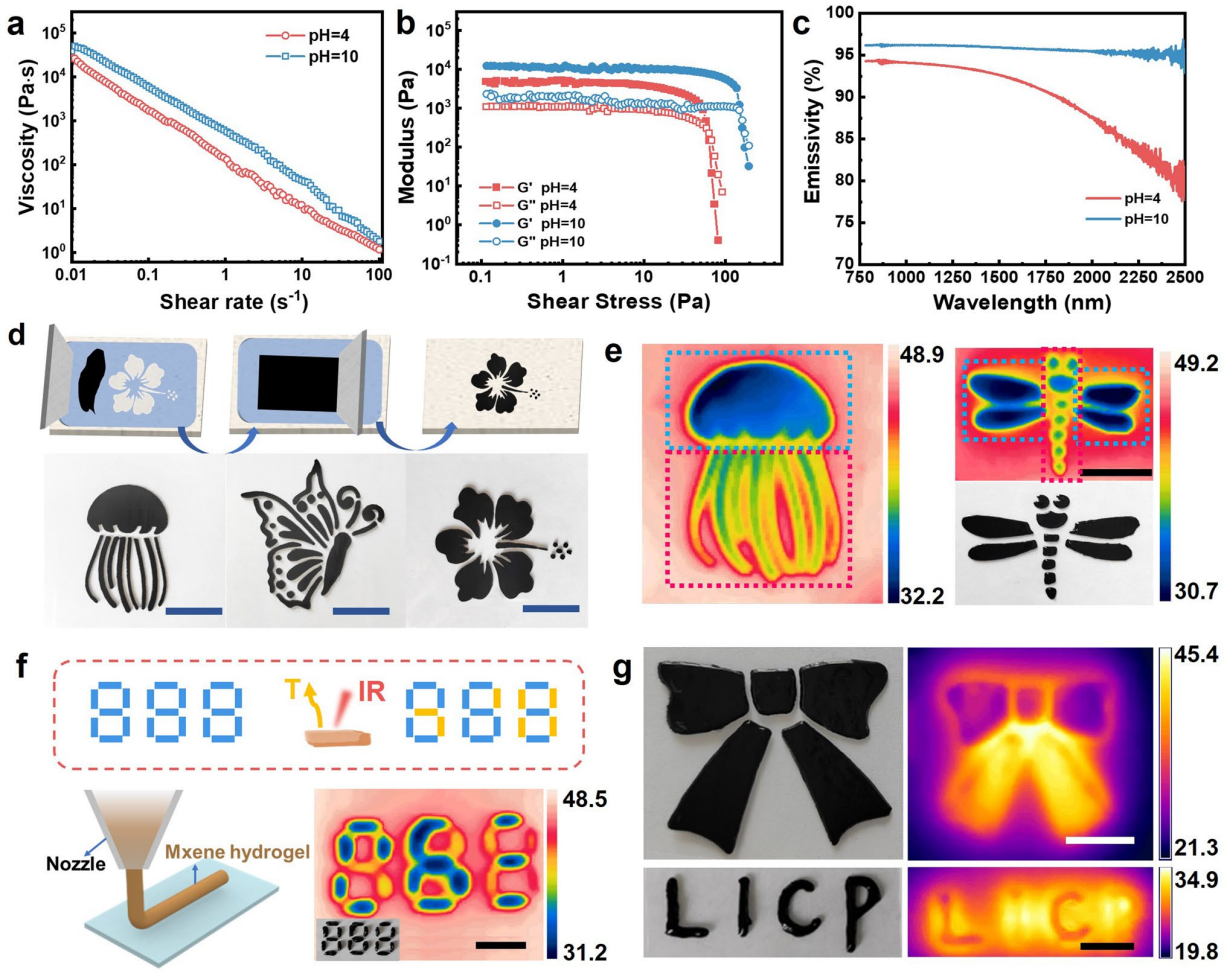
Rheological property, ink printing and anticounterfeiting applications of the MXene gel. **a** Viscosity of the Ti_3_C_2_T_*x*_ gels in a shear rate from 0.01 to 100 s^−1^. **b** *G*′ and *G*′′ of the gels as a function of shear stress. **c** Emissivity spectra of the gels in the NIR band (750–2500 nm). **d** Screen-printing process and screen-printed patterns of the MXene gel inks. **e** Security jellyfish and dragonfly patterns for photoemissive anticounterfeiting. The blue and red rectangular boxes indicate the areas covered by the pH 4 and 10 gels, respectively. **f** Schematic and photographic illustrations of the photothermal encryption using the passwords from extrusion-printing of the MXene gels. **g** Extrusion-printed anticounterfeiting patterns before (left) and after (high) exposure to 3 W cm^−2^ NIR irradiations. Scale bar = 1 cm. (Color figure online)

Figure 5a depicts a typical shear-thinning behavior of the pH 4 and 10 Ti_3_C_2_T_*x*_ gels, which is indispensable for continuous extrusion through screen meshes as inks [63]. Figure 5b displays a good viscoelasticity (*G′* > 5 kPa and *G′′* > 1 kPa), large *G′*/*G′′* ratio (~ 5.3 for pH 4 and ~ 11.3 for pH 10) and high yield stress (~ 49 Pa for pH 4 and ~ 138 Pa for pH 10) of the gels that allow rapid and high-precision screen- or extrusion-printing into various high-resolution and complicated patterns [35]. Together with their impressive thixotropic property (Fig. 1f), these gels were ideal candidates for direct ink writing, high-viscosity printing and other liquid processing techniques. Figures 5c and S26 illustrate a higher near-infrared (NIR) emissivity at high wavelength and light-to-heat conversion efficiency of the pH 10 MXene gel relative to those of the pH 4 gel, which can be exploited to produce temperature differences under thermal imaging and consequently colorful screen- or extrusion-printed patterns prospective for IR camouflage and anti-counterfeiting applications [9, 35, 64]. The higher emissivity and light-to-heat transition efficiency were likely owing to the relatively low content of –F and –OH terminations at pH 10 and interconnected MXene architectures acting as crumpled or hierarchical textures with multiple light scattering and trapping [9, 65].

Figure 5d shows the design and screen-printing process of the gel-like MXene ink, and well-defined, high-resolution patterns including jellyfish, butterfly, flower and dragonfly (Fig. 5e) printed from a binary mixture of the pH 4 and 10 gels. Figure S27 demonstrates that the gel ink could tightly and flexibly adhere onto a polyethylene terephthalate (PET) substrate used for screen-printing. After placing onto an external heating plate, significant color differences appeared on the pristine black composite patterns, in which the pH 4 gel-covered regions emitted cool color and the pH 10 gel-coating parts exhibited warm color under a thermal imager (Fig. 5e). As a consequence, the anticounterfeited jellyfish and dragonfly patterns decoding the encrypted messages can be detected in the thermal images after heating (Fig. 5e). The high yield stress and good thixotropy also allowed high-precision extrusion-printing of the gels into patterns of numbers, bowknot and abbreviation of our institute (i.e., “LICP”) as schematically and photographically illustrated in Figs. 5f, g and S28. After exposure to external NIR irradiations, the anticounterfeited passwords or patterns can be recognized by thermal imaging building on the prominent color differences between the low- and high-temperature regions covered by pH 4 and 10 MXene gels (Fig. 5f, g), respectively. To eliminate any interferences from insufficient heat transfer or light absorption, all the gel ink coatings were heated or irradiated for abundant time of at least 10 min. Our results therefore may indicate a new design strategy for developing functional gel-like MXene inks capable of achieving both photoemissive and photothermal security and encryption.

So far, MXene-based IR anti-counterfeiting and thermal camouflage usually rely on different amounts of surface coatings that are able to effectively distinguish the light-emissive or light-to-heat transition efficiency of resulting 3D MXene architectures under external heating or IR irradiations [9, 11, 35, 65]. Herein, taking advantages of mild centrifugation leading to gel-like MXene inks with excellent rheological properties and the pH-controlled internal self-assembly of Ti_3_C_2_T_*x*_ nanosheets resulting in distinct photoemissive and photothermal-conversion efficacies, our study may provide new paradigms toward high-performance, low-cost and eco-friendly MXene inks that are anticipated to have good prospects in modern military, information encryption and anticounterfeiting fields [9, 66]. Together with their robust and controlled lubricity and ultrahigh capacitance and good longevity, we envision that the versatile MXene gels may find a diverse range of potential applications in materials science, nanotechnology, green tribology and mechanical engineering, by contrast to some of MXene-based architectures restricted to a specific purpose.

In previous work, we have fabricated a centrifugation-induced graphene oxide gel displaying responsive lubrication and both mechanical and tribological adaptivity building on similar pH-controlled internal structural changes, which can not only act as a new type of smart and green semi-solid lubricant but also provide guidance for design and creation of novel purely inorganic-based biomimetic soft materials [20]. In this study, taking advantages of the excellent electrical conductivity, high specific capacitance and tunable light-emissive and photothermal-conversion properties of the MXene gelator, a centrifugation-triggered inorganic gel capable of functioning as supercapacitor electrodes and NIR-based anticounterfeiting inks in addition to pH-controlled lubricant has been produced. It may further expand the potential applications of such centrifugation-assisted gelated semi-solid material in the fields of energy storage, screen- and/or extrusion-printing, thermal camouflage and security coding.

## Conclusions

We have demonstrated a “proof-of-concept” of creating high-water-content MXene gels thoroughly free of any linkers using brief and moderate centrifugation. Such simple and low-cost strategy could induce an effective spontaneous gelation of Ti_3_C_2_T_*x*_ MXene nanosheets with a very low dispersion concentration of 0.5 mg mL^−1^. The resultant gels possessed high viscoelasticity, large yield stress and excellent thixotropic performance that were applicable to high-precision screen- or extrusion-printing into multifarious high-resolution, complicated patterns. On the basis of acid/alkaline-triggered variations in the surface terminations of MXene nanosheets and consequently topological reconfiguration in their internal structures, the gels expressed low CoF and abrasion related to potent lubrication and remarkable specific capacitances promising for advanced supercapacitors at relatively low pH values (e.g., 4), while relatively high friction necessary for maintaining desired efficiency, accuracy and reliability of machines and devices as well as favorable and durable capacitances useful for developing long-life energy storage materials at high pH values (e.g., 10). Their distinct NIR emissivity and light-to-heat transition efficiency at pH 4 and 10 can be utilized to achieve both photoemissive and photothermal security and encryption, which showed good prospects in information encryption and anticounterfeiting fields. Therefore, our study may provide new insights into developing facilely prepared, low-expense, high-performance and multifunctional MXene-based soft materials that are anticipated to find extensive applications in materials science, colloidal chemistry, nanotechnology, tribology and mechanical engineering.

## Supplementary Information

Below is the link to the electronic supplementary material.

Supplementary file1 (PDF 1609 KB)
