# Supplementary material for: Versatile MXene Gels Assisted by Brief and Low-Strength Centrifugation
Source: Nanomicro Lett. 2024 Jan 22;16:94. doi: 10.1007/s40820-023-01302-3 (PMC10803715; doi:10.1007/s40820-023-01302-3)
Supplement: Supplementary file 1 — Supplementary file1 (PDF 1609 KB) [file 40820_2023_1302_MOESM1_ESM.pdf]

Supplementary Information for

# Versatile MXene Gels Assisted by Brief and Low-Strength Centrifugation

Weiyan Yu[1,2], Yi Yang[1,2], Yunjing Wang[2], Lulin Hu[1,2], Jingcheng Hao[2,3,]*, Lu Xu[1,2,]* and Weimin Liu[1,2]

[1] State Key Laboratory of Solid Lubrication, Lanzhou Institute of Chemical Physics, Chinese Academy of Sciences, Lanzhou 730000, P. R. China

[2] Shandong Laboratory of Advanced Materials and Green Manufacturing at Yantai, Yantai 264006, P. R. China

[3] Key Laboratory of Colloid and Interface Chemistry & Key Laboratory of Special Aggregated Materials, Shandong University, Jinan 250100, P. R. China

*Corresponding authors. E-mail: jhao@sdu.edu.cn (Jingcheng Hao); xulu@licp.cas.cn (Lu Xu)

## Supplementary Figures and Tables

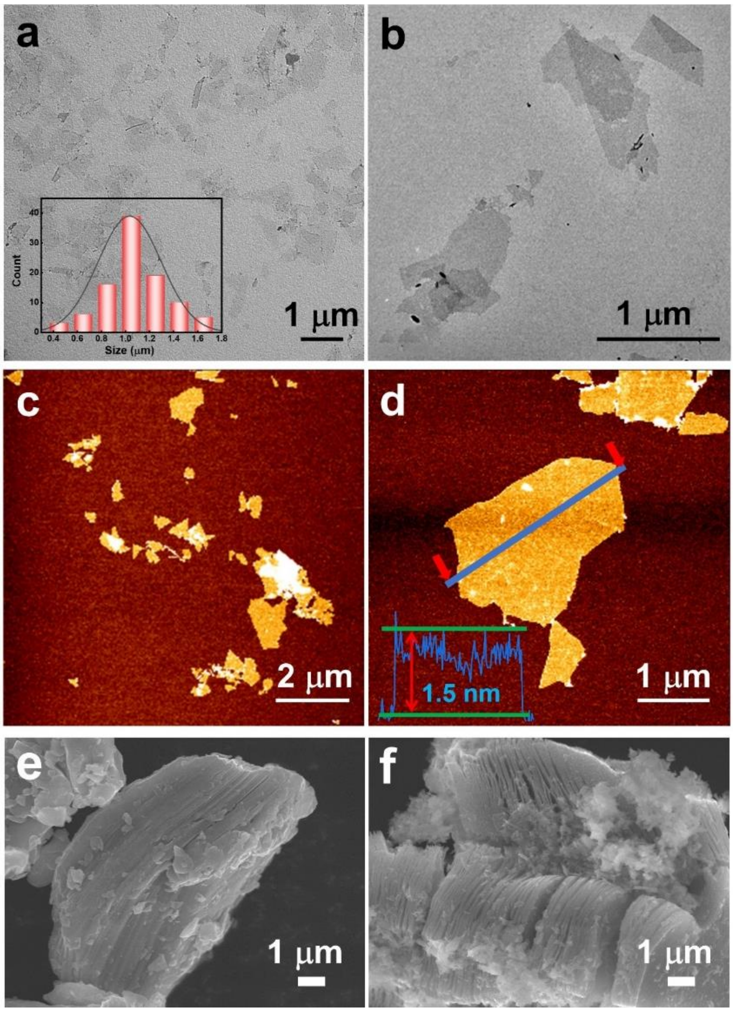


**Fig. S1 a, b** TEM and **c, d** AFM observations of exfoliated ultrathin $Ti_3C_2T_x$ MXene. SEM images of **e** pristine $Ti_3AlC_2$ MAX phase and **f** multilayered $Ti_3C_2T_x$ MXene obtained after etching. Insets in **a** and **d** are the particle size distribution and heigh profile of the MXene nanosheets

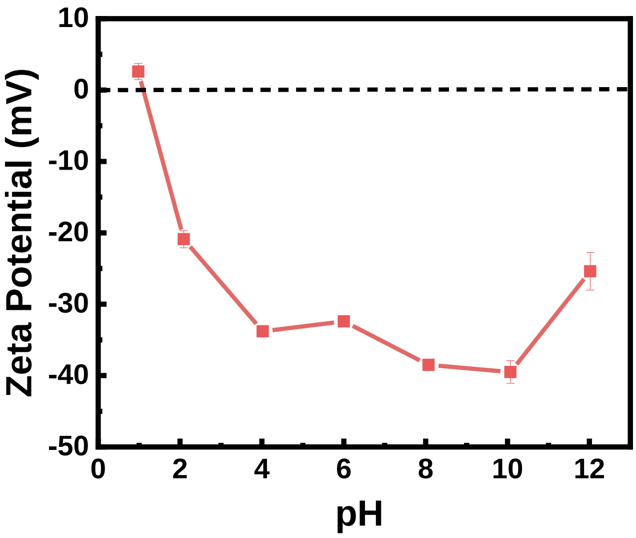


**Fig. S2** Zeta potential measurements of $Ti_3C_2T_x$ dispersions at different pH values. T = 25 °C

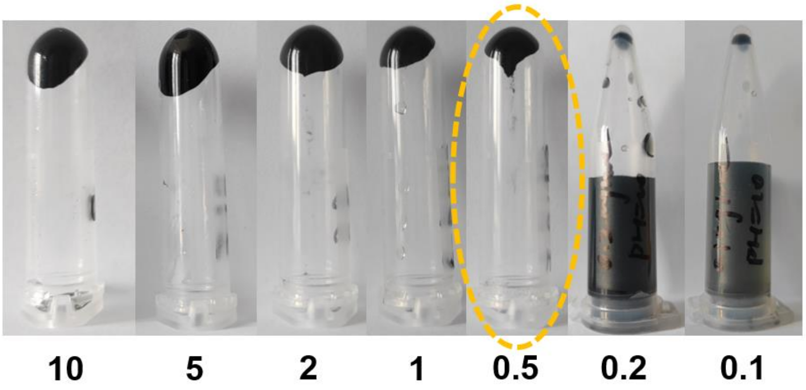


**Fig. S3** Photographs of pH 10 $Ti_3C_2T_x$ dispersions at different particle concentrations (in mg $mL^{-1}$) after centrifugation at 10000 ×g for 30 s. T = 25 °C

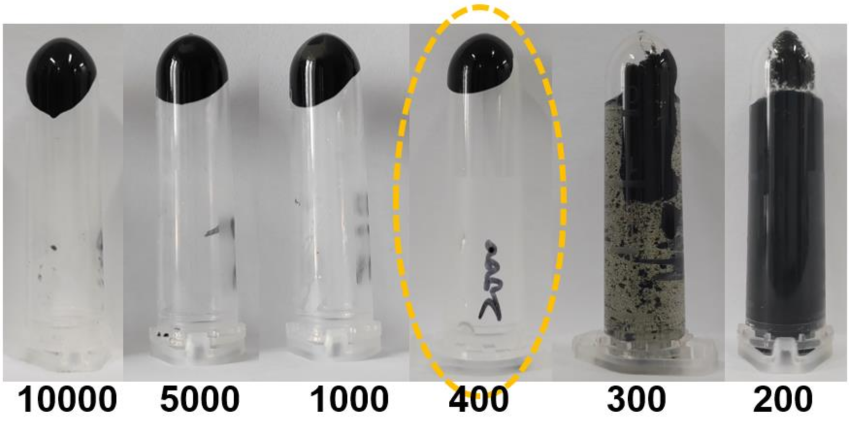


**Fig. S4** Photographs of pH 10, 0.5 mg $mL^{-1}$ $Ti_3C_2T_x$ dispersions after exposure to different relative centrifugal forces (RCFs, in ×g) for 30 s. T = 25 °C

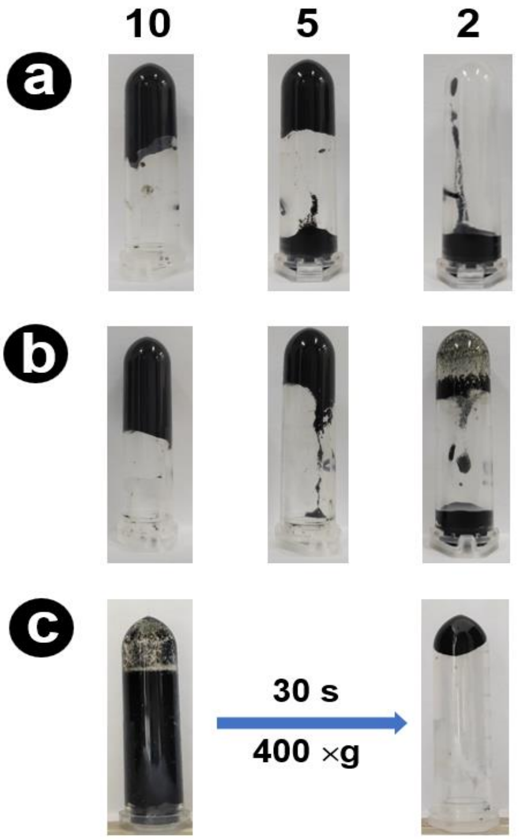


**Fig. S5** Photographs of pH 10 $Ti_3C_2T_x$ dispersions at different particle concentrations (in mg $mL^{-1}$) after addition of **a** $Fe^{2+}$ and **b** $Zn^{2+}$ to a final mass ratio of 3:8 (metal chloride-to-MXene). **c** Photographs of an aqueous dispersion containing 2 mg $mL^{-1}$ $Ti_3C_2T_x$ nanosheets and 5 mmol $L^{-1}$ $Fe^{2+}$ before and after exposure to 400 ×g centrifugation for 30 s. T = 25 °C

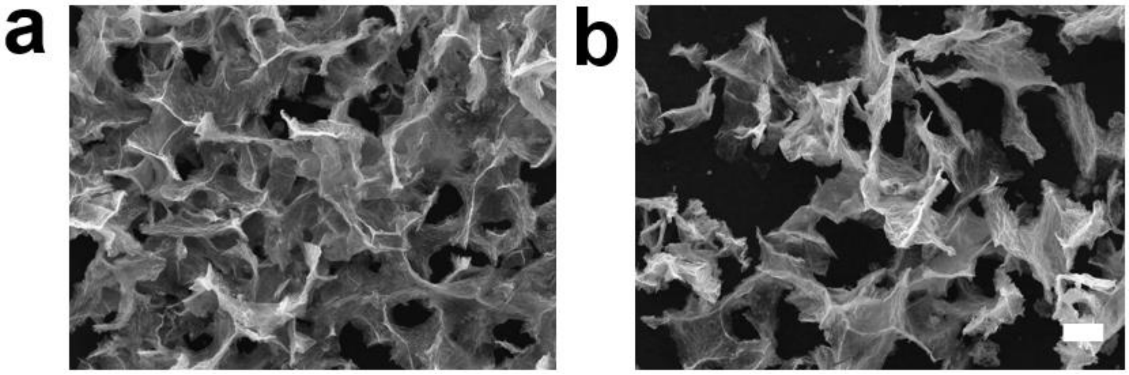


**Fig. S6** SEM observations of freeze-dried pH 10 MXene gels **a** and dispersions **b**. Scale bar = 20 μm

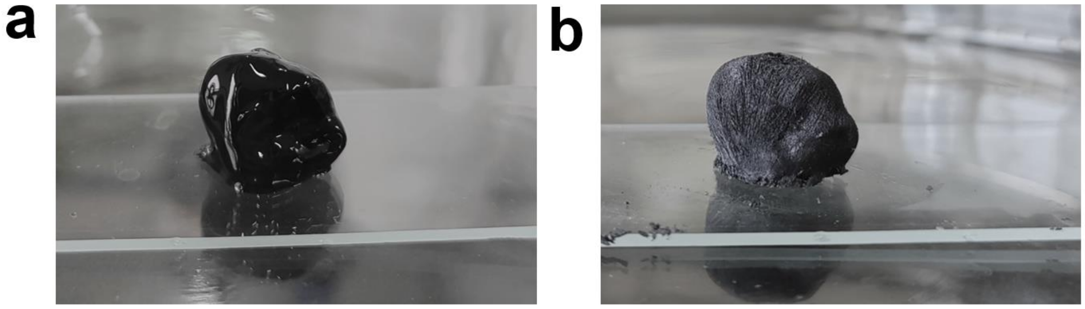


**Fig. S7** 3D macrostructures of a pH 10 MXene gel **a** before and **b** after complete lyophilization

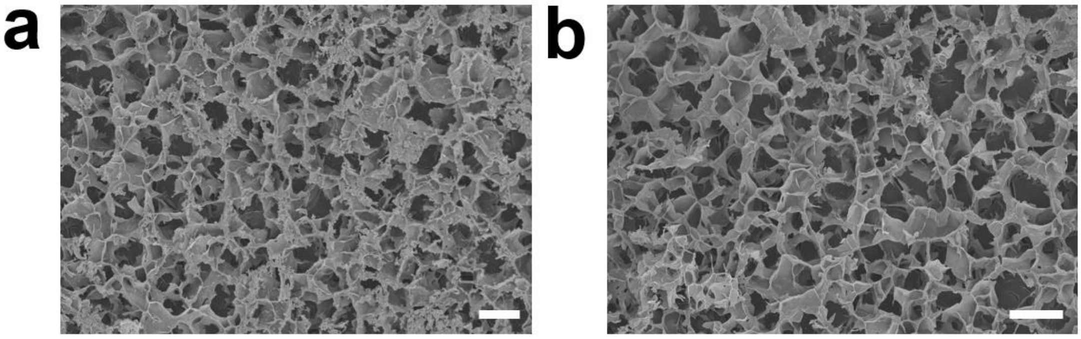


**Fig. S8** Cryogenic SEM observations on a pristine pH 10 MXene gel. Scale bar = 5 μm

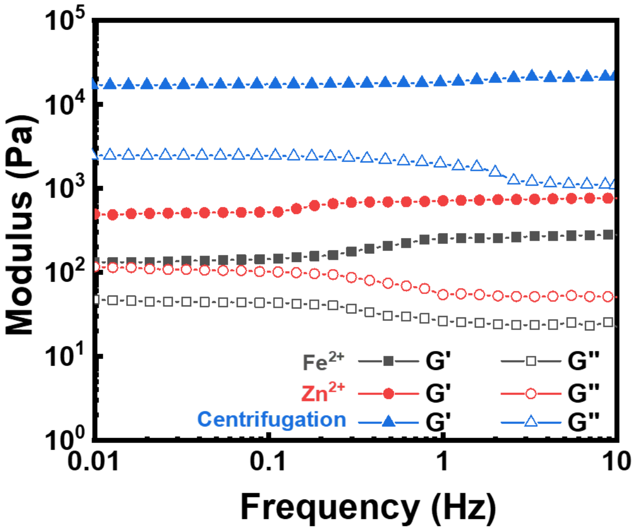


**Fig. S9** Comparison in viscoelasticity between the $Ti_3C_2T_x$ gels with an identical water content of ~98 wt% triggered by centrifugation and divalent metal ions. T = 25 °C

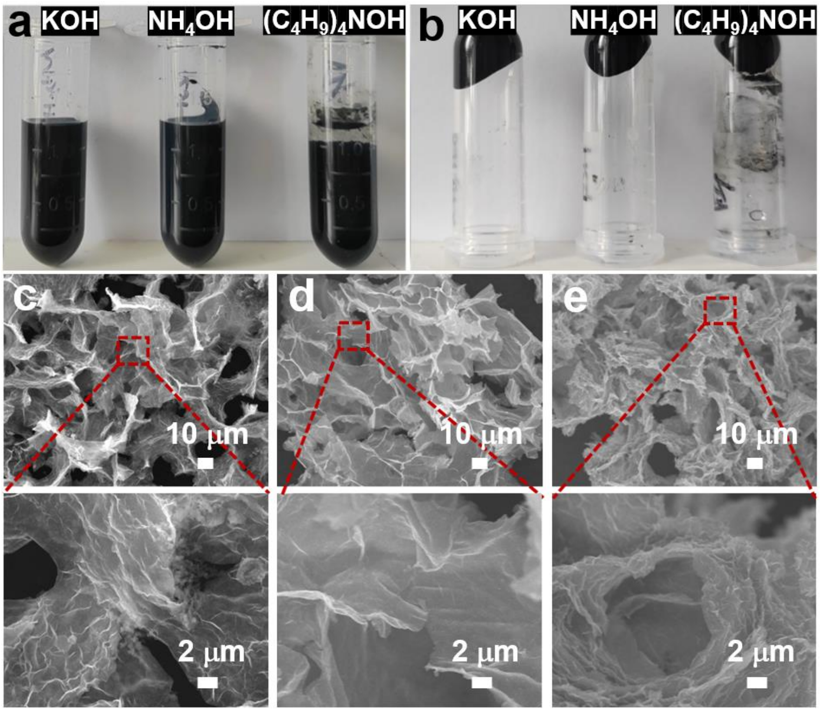


**Fig. S10** Photographs of 10 mg mL$^{-1}$, pH 10 MXene dispersions prepared with different alkalis **a** before and **b** after centrifugation at 400 ×g for 30 s. SEM images of the centrifugation-assisted MXene gels prepared with **c** KOH, **d** $NH_4OH$ and **e** $(C_4H_9)_4NOH$. T = 25 °C

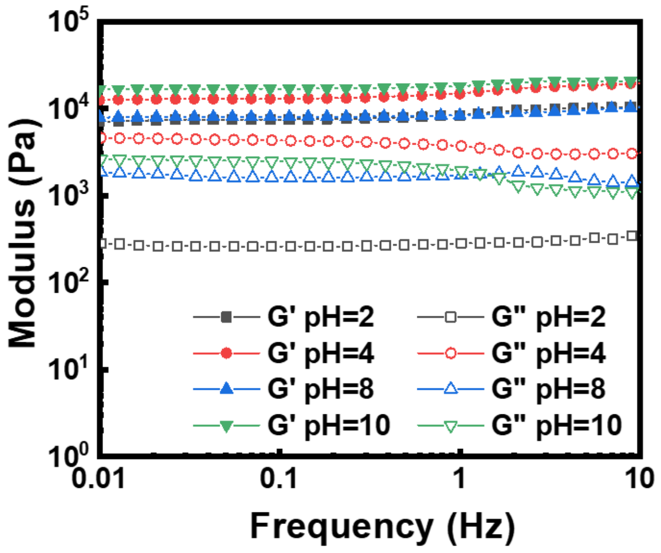


**Fig. S11** Rheological property of the centrifugation-assisted MXene gel with different internal pH values. T = 25 °C

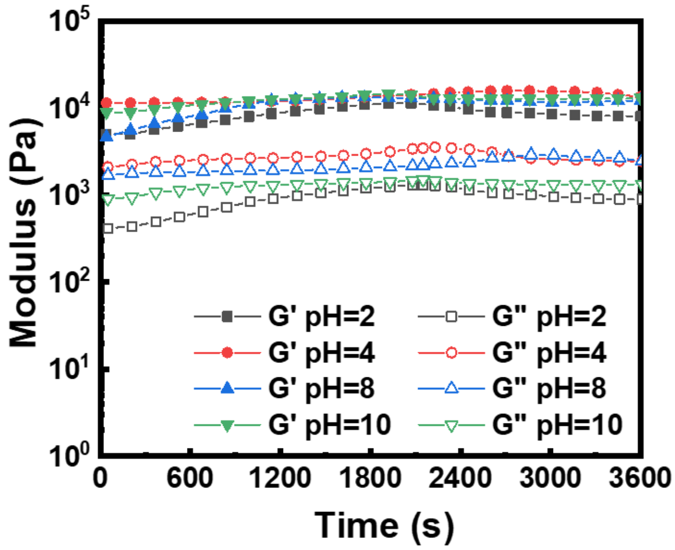


**Fig. S12** Changes in viscoelasticity of the MXene gels over time at a constant shear frequency and strain of 1 Hz and 5%, respectively. T = 25 °C

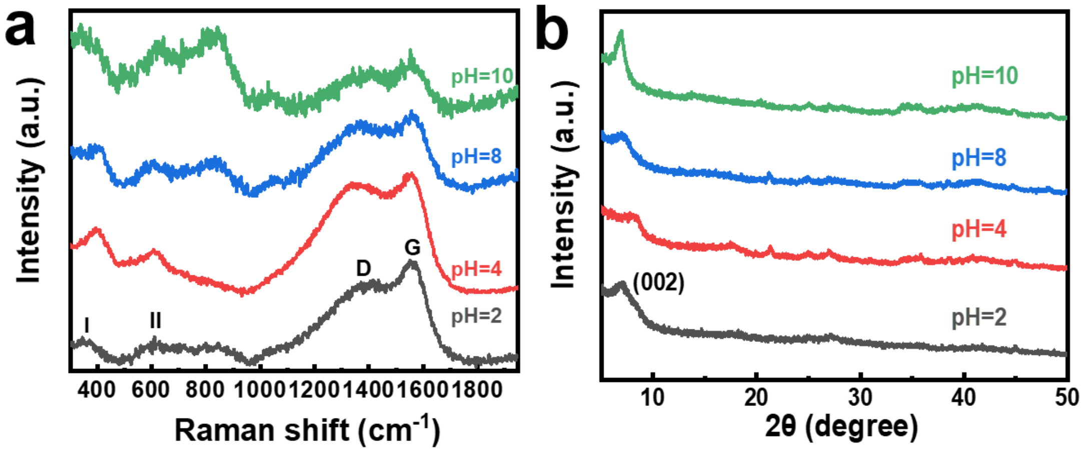


**Fig. S13 a** Raman and **b** XRD profiles of lyophilized MXene gels at different pH values. T = 25 °C

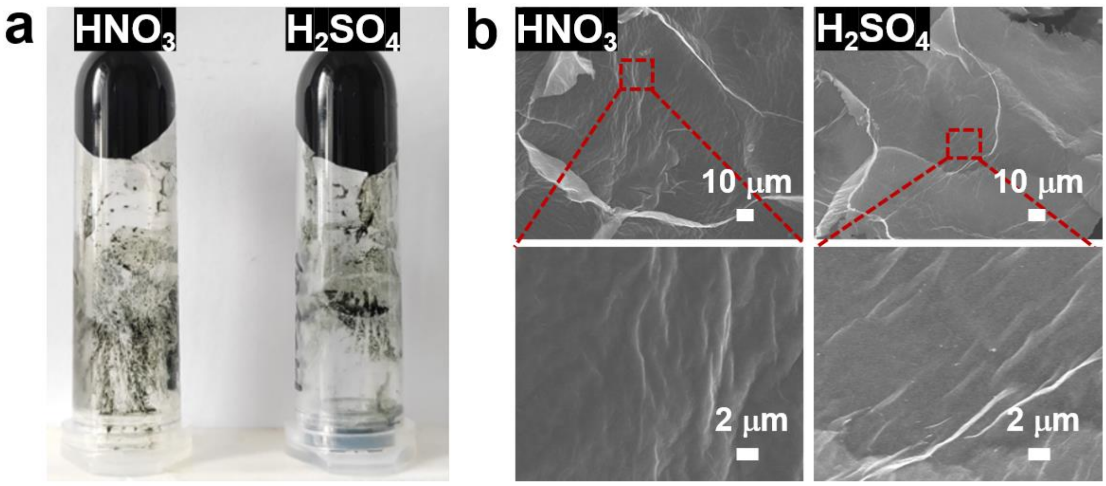


**Fig. S14 a** Photographs and **b** internal microstructures of pH 2 MXene gels prepared using $H_2SO_4$ and $HNO_3$. T = 25 °C

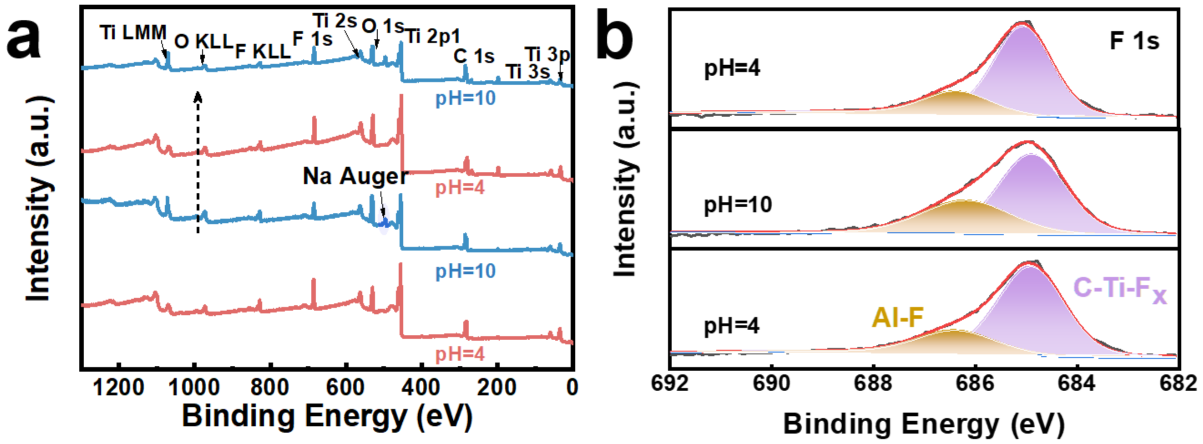


**Fig. S15 a** Full XPS and **b** XPS F 1s spectra of a lyophilized MXene gel after alternately changing pH between 4 and 10. T = 25 °C

**Table S1** Contents of each terminal group determined by the deconvolution and integration of corresponding band areas from the XPS profiles

| **pH** | **-F** | **-O** | **-OH** |
|---|---|---|---|
| pH=4 | 50.4% | 23.9% | 25.7% |
| pH=10 | 30.3% | 48.0% | 21.7% |
| pH=4 | 48.2% | 24.0% | 27.8% |

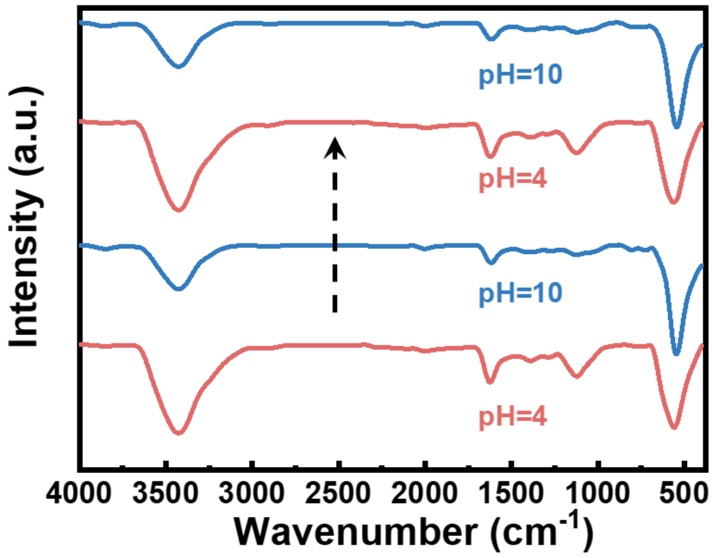


**Fig. S16** FTIR profile of a freeze-dried MXene gel after alternately changing its internal pH value between 4 and 10. T = 25 °C

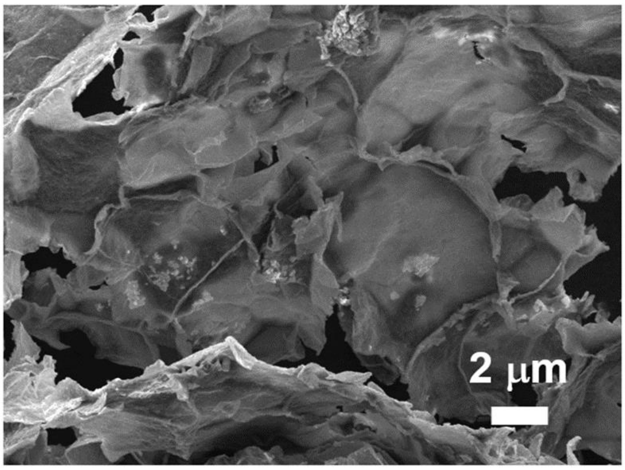


**Fig. S17** Internal microstructure of a pH 4 gel after addition of an equal amount of NaCl instead of NaOH

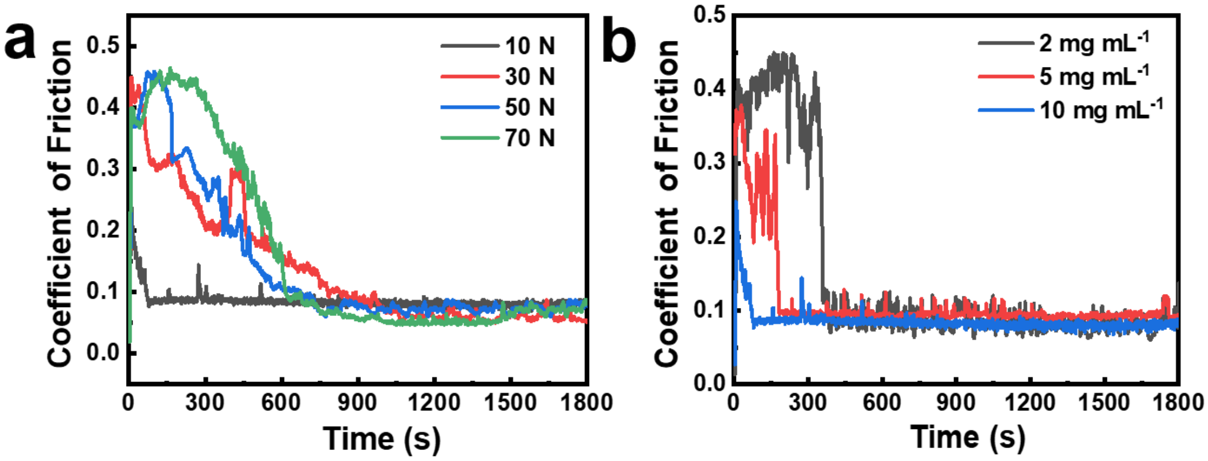


**Fig. S18** Effect of **a** applied normal load and **b** initial particle concentration on the CoF of a pH 8 MXene gel. Sliding velocity = 10 mm $s^{-1}$. T = 25 °C

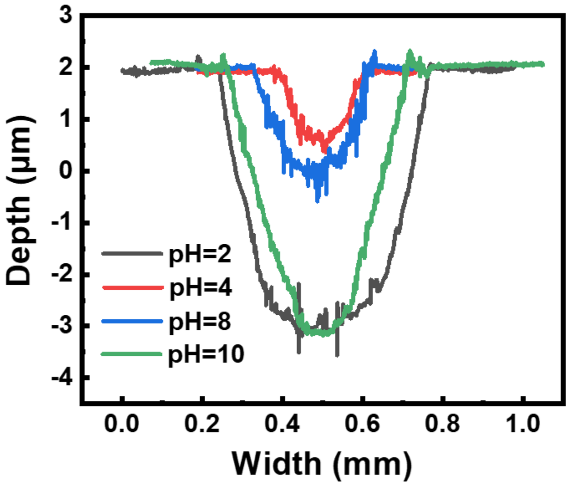


**Fig. S19** Width and depth profiles of the wear scar on a steel substrate lubricated with MXene gels in different pH values

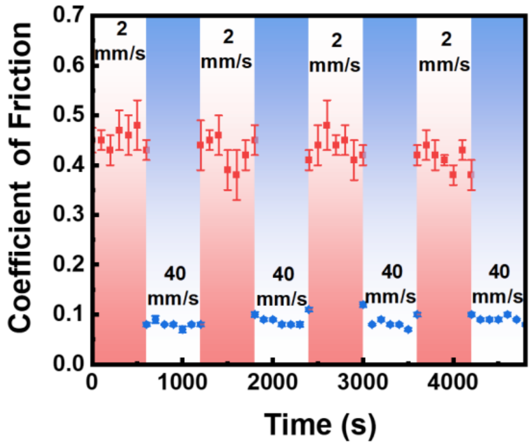


**Fig. S20** Variations in the CoF of a pH 4 MXene gel upon alternately changing sliding velocity between 2 and 40 mm $s^{-1}$. T = 25 °C

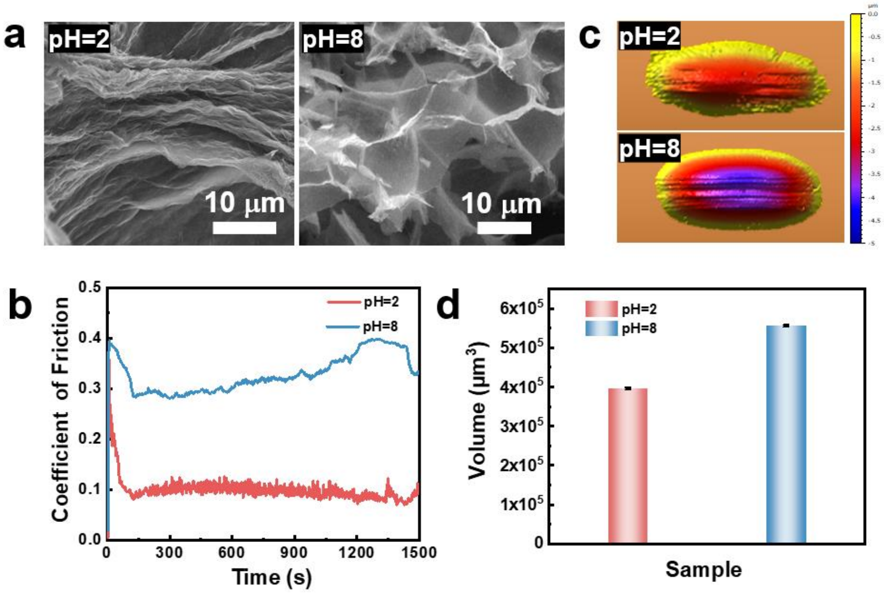


**Fig. S21** **a** Internal self-assembled structure and **b** CoF of the MXene-PDDA composite gel at pH 2 and 8. **c** 3D surface topography and **d** abrasive volume of a steel substrate lubricated with the PDDA-containing gel at pH 2 and 8 under 10 N and 10 mm $s^{-1}$. T = 25 °C

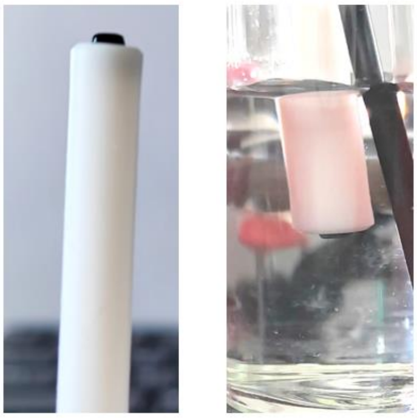

**Fig. S22** Photographs of an electrode fabricated with the $Ti_3C_2T_x$ gel

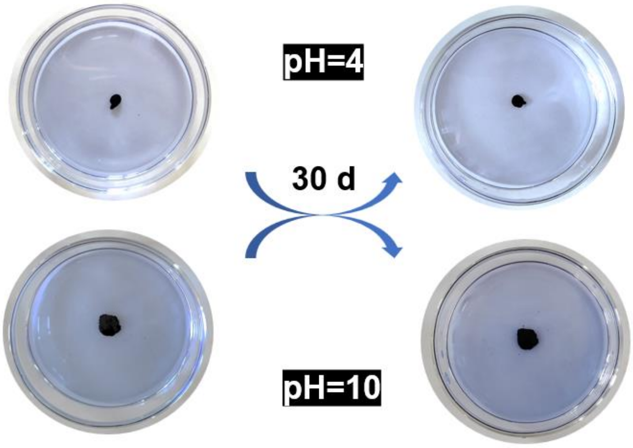


**Fig. S23** Photographs of the pH 4 and 10 $Ti_3C_2T_x$ gels after immersed in water reservoirs stained with dye methylene blue for better visualization for 30 days. T = 25 °C

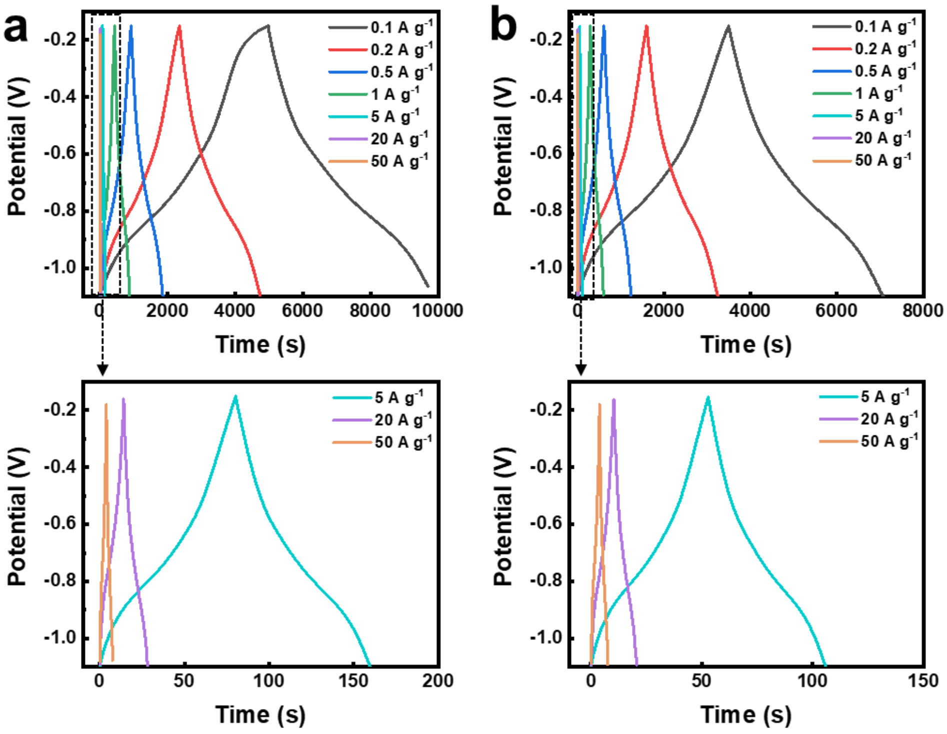


**Fig. S24** GCD curves of **a** pH 4 and **b** 10 MXene gels at current densities between 0.1 and 50 A $g^{-1}$. T = 25 °C

**Table S2** Electrochemical performances of the MXene gel-based electrodes reported previously

| Gels | Electrolyte | Potential (V) | Specific capacitance (F $g^{-1}$) | Cyclic stability | Refs. |
|---|---|---|---|---|---|
| MXene/$Fe^{2+}$ | 3 M $H_2SO_4$ | -1.1 to -0.15 ($Hg/Hg_2SO_4$) | ~270 (10 mV $s^{-1}$)<br>~255 (100 mV $s^{-1}$) | 97.1% after 10,000 cycles | [S1] |
| MXene/HA | 3 M $H_2SO_4$ | -1.1 to -0.15 ($Hg/Hg_2SO_4$) | ~255 (10 mV $s^{-1}$)<br>~90 (100 mV $s^{-1}$) | 91.7% after 10,000 cycles | [S2] |
| MXene/$H_2SO_4$ hydrogel film | 3 M $H_2SO_4$ | -1.1 to -0.1 ($Hg/Hg_2SO_4$) | ~375 (10 mV $s^{-1}$) | 90% after 10,000 cycles | [S3] |
| $H_2SO_4$-thawed MXene | 3 M $H_2SO_4$ | -1.2 to -0.2 ($Hg/Hg_2SO_4$) | ~393 (5 mV $s^{-1}$) | 95.5% after 10,000 cycles | [S4] |
| MXene/rGO hydrogel film | 3 M $H_2SO_4$ | -1.1 to -0.15 ($Hg/Hg_2SO_4$) | ~300 (10 mV $s^{-1}$)<br>~280 (100 mV $s^{-1}$) | 94.3% after 10,000 cycles | [S5] |
| MXene/GO | 3 M $H_2SO_4$ | -0.5 to 0.3 (Ag/AgCl) | ~470 (10 mV $s^{-1}$)<br>~380 (100 mV $s^{-1}$) | ~98% after 8,000 cycles | [S6] |
| MXene/$Al^{3+}$ | 1 M $H_2SO_4$ | -0.4 to 0.3 (Ag/AgCl) | ~275 (100 mV $s^{-1}$) | ~90% after 5,000 cycles | [S7] |
| MXene/rGO/CNT | 3 M $H_2SO_4$ | -0.6 to 0.25 (Ag/AgCl) | ~300 (100 mV $s^{-1}$) | 97.1% after 10000 cycles | [S8] |
| $Zn^{2+}$/MXene hydrogel film | 1 M $H_2SO_4$ | -0.6 to 0.2 (Ag/AgCl) | ~390 (10 mV $s^{-1}$)<br>~350 (100 mV $s^{-1}$) | ~98% after 10,000 cycles | [S9] |
| MXene | 3 M $H_2SO_4$ | -1.1 to -0.15 ($Hg/Hg_2SO_4$) | pH 4:<br>~635 (5 mV $s^{-1}$)<br>~604 (10 mV $s^{-1}$)<br>~408 (100 mV $s^{-1}$)<br>pH 10:<br>~344 (5 mV $s^{-1}$)<br>~322 (10 mV $s^{-1}$)<br>~305 (100 mV $s^{-1}$) | pH 4:<br>85.9% after 10,000 cycles<br>pH 10:<br>96.7% after 10,000 cycles | This work |

**Table S3** Electrical conductivity of previously reported MXene-based aerogels

| Aerogels | Electrical conductivity (S $m^{-1}$) | Refs. |
|---|---|---|
| MXene/CNF | 1.8 | [S10] |
| MXene/rGO | 695.9 | [S11] |
| MXene/rGO/CNT | 9-92 | [S8] |
| MXene/CNF/CNT | 2400 | [S12] |
| MXene/polyimide | 4 | [S13] |
| MXene/silver nanowire | 1532 | [S14] |
| MXene/acidified CNT | 447 | [S15] |
| MXene/rGO | 36.2 | [S16] |
| MXene | 20400 (pH 4)<br>3800 (pH 10) | This work |

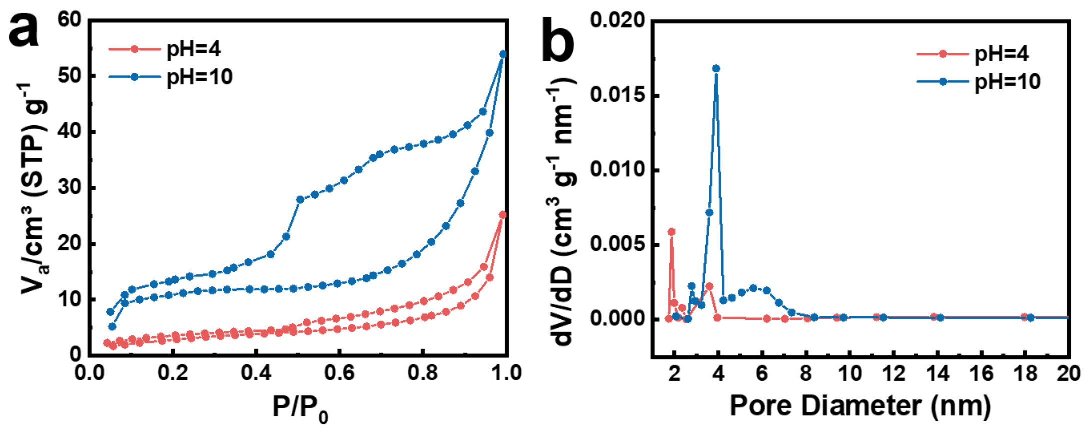


**Fig. S25 a** $N_2$ adsorption/desorption isotherms and **b** pore size distribution of lyophilized $Ti_3C_2T_x$ gels at 25 °C

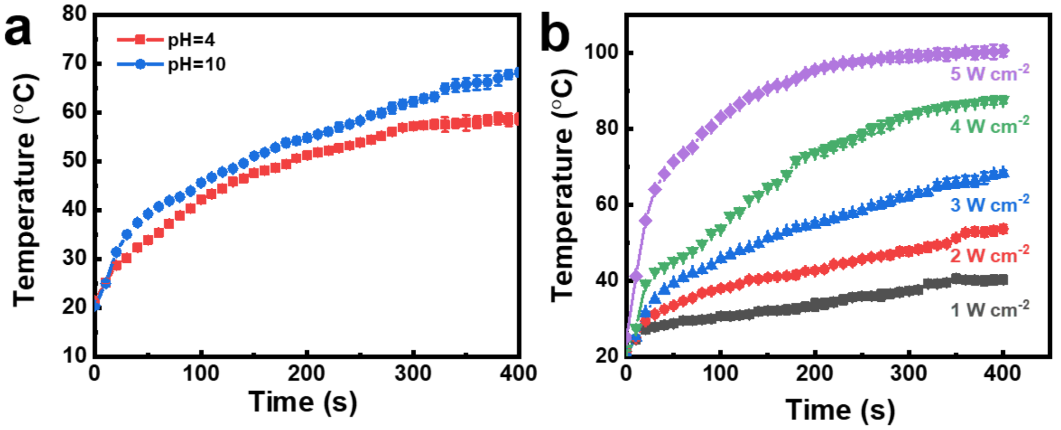


**Fig. S26 a** Temperature variations of the $Ti_3C_2T_x$ gels after irradiated by a 3 W $cm^{-2}$ NIR light for 400 s. **b** Effect of irradiation power on the temperature increment of a pH 10 $Ti_3C_2T_x$ gel. It can be seen that a power above 3 W $cm^{-2}$ would cause a relatively abrupt temperature elevation that may lead to severe water evaporation and changes in the gel properties

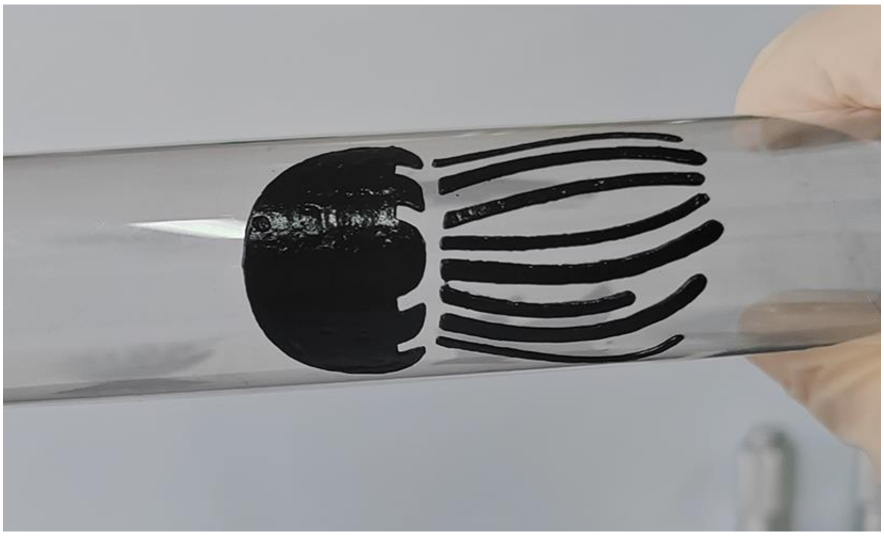

**Fig. S27** Photograph of the jellyfish-shaped MXene gel coating on a PET substrate in the bending state

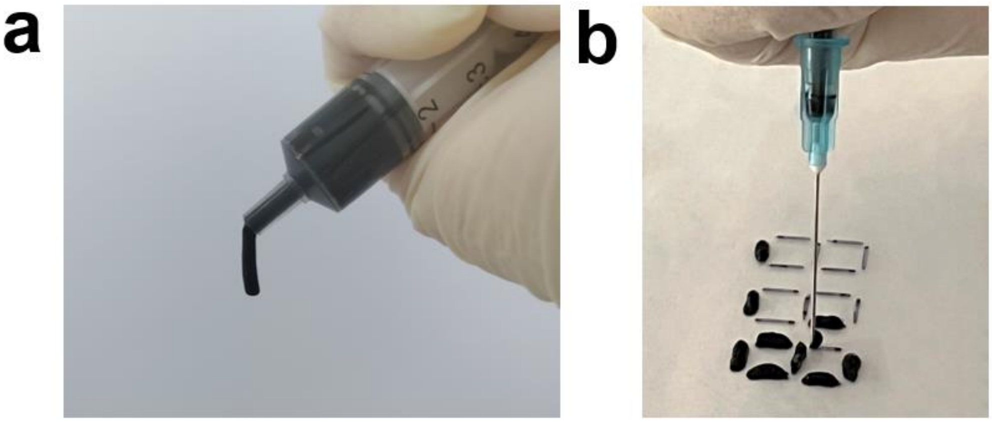


**Fig. S28** Photographs of **a** extrusion of the gel through a needle syringe and **b** extrusion-printing of the MXene gel into anti-counterfeiting passwords

## Supplementary References

[S1] Y. Deng, T. Shang, Z. Wu, Y. Tao, C. Luo et al., Fast gelation of $Ti_3C_2T_x$ MXene initiated by metal ions. Adv. Mater. **31**, 1902432 (2019). https://doi.org/10.1002/adma.201902432

[S2] Z. Wu, Y. Deng, J. Yu, J. Han, T. Shang et al., Hydroiodic-acid-initiated dense yet porous $Ti_3$ $C_2$ $t_x$ MXene monoliths toward superhigh areal energy storage. Adv. Mater. **35**, e2300580 (2023). https://doi.org/10.1002/adma.202300580

[S3] M.R. Lukatskaya, S. Kota, Z. Lin, M.-Q. Zhao, N. Shpigel et al., Ultra-high-rate pseudocapacitive energy storage in two-dimensional transition metal carbides. Nat. Energy **2**, 17105 (2017). https://doi.org/10.1038/nenergy.2017.105

[S4] X. Huang, J. Huang, D. Yang, P. Wu, A multi-scale structural engineering strategy for high-performance MXene hydrogel supercapacitor electrode. Adv. Sci. **8**, e2101664 (2021). https://doi.org/10.1002/advs.202101664

[S5] Z. Wu, X. Liu, T. Shang, Y. Deng, N. Wang et al., Reassembly of MXene hydrogels into flexible films towards compact and ultrafast supercapacitors. Adv. Funct. Mater. **31**, 2102874 (2021). https://doi.org/10.1002/adfm.202102874

[S6] P. Dutta, A. Sikdar, A. Majumdar, M. Borah, N. Padma et al., Graphene aided gelation of MXene with oxidation protected surface for supercapacitor electrodes with excellent gravimetric performance. Carbon **169**, 225–234 (2020). https://doi.org/10.1016/j.carbon.2020.07.041

[S7] Z. Zhang, Z. Yao, Y. Li, S. Lu, X. Wu et al., Cation-induced Ti3C2Tx MXene hydrogel for capacitive energy storage. Chem. Eng. J. **433**, 134488 (2022). https://doi.org/10.1016/j.cej.2021.134488

[S8] X. Yang, Y. Yao, Q. Wang, K. Zhu, K. Ye et al., 3D macroporous oxidation-resistant $Ti_3C_2T_x$ MXene hybrid hydrogels for enhanced supercapacitive performances with ultralong cycle life. Adv. Funct. Mater. **32**, 2109479 (2022). https://doi.org/10.1002/adfm.202109479

[S9] T. Yun, G.S. Lee, J. Choi, H. Kim, G.G. Yang et al., Multidimensional $Ti_3C_2T_x$ MXene architectures *via* interfacial electrochemical self-assembly. ACS Nano **15**, 10058–10066 (2021). https://doi.org/10.1021/acsnano.1c01727

[S10] N. Wu, Y. Yang, C. Wang, Q. Wu, F. Pan et al., Ultrathin cellulose nanofiber assisted ambient-pressure-dried, ultralight, mechanically robust, multifunctional MXene aerogels. Adv. Mater. **35**, e2207969 (2023). https://doi.org/10.1002/adma.202207969

[S11] S. Zhao, H.-B. Zhang, J.-Q. Luo, Q.-W. Wang, B. Xu et al., Highly electrically conductive three-dimensional $Ti_3C_2T_x$ MXene/reduced graphene oxide hybrid aerogels with excellent electromagnetic interference shielding performances. ACS Nano **12**, 11193–11202 (2018). https://doi.org/10.1021/acsnano.8b05739

[S12] T. Xu, Q. Song, K. Liu, H. Liu, J. Pan et al., Nanocellulose-assisted construction of multifunctional MXene-based aerogels with engineering biomimetic texture for pressure sensor and compressible electrode. Nano-Micro Lett. **15**, 98 (2023). https://doi.org/10.1007/s40820-023-01073-x

[S13] J. Liu, H.-B. Zhang, X. Xie, R. Yang, Z. Liu et al., Multifunctional, superelastic, and lightweight MXene/polyimide aerogels. Small **14**, e1802479 (2018). https://doi.org/10.1002/smll.201802479

[S14] H. Liu, Z. Huang, T. Chen, X. Su, Y. Liu et al., Construction of 3D MXene/Silver nanowires aerogels reinforced polymer composites for extraordinary electromagnetic interference shielding and thermal conductivity. Chem. Eng. J. **427**, 131540 (2022). https://doi.org/10.1016/j.cej.2021.131540

[S15] Z. Deng, P. Tang, X. Wu, H.-B. Zhang, Z.-Z. Yu, Superelastic, ultralight, and conductive $Ti_3C_2T_x$ MXene/acidified carbon nanotube anisotropic aerogels for electromagnetic interference shielding. ACS Appl. Mater. Interfaces **13**, 20539–20547 (2021). https://doi.org/10.1021/acsami.1c02059

[S16] D. Jiang, J. Zhang, S. Qin, Z. Wang, K.A.S. Usman et al., Superelastic $Ti_3C_2T_x$ MXene-based hybrid aerogels for compression-resilient devices. ACS Nano **15**, 5000–5010 (2021). https://doi.org/10.1021/acsnano.0c09959
